# MHC matching fails to prevent long-term rejection of iPSC-derived neurons in non-human primates

**DOI:** 10.1038/s41467-019-12324-0

**Published:** 2019-09-25

**Authors:** Romina Aron Badin, Aurore Bugi, Susannah Williams, Marta Vadori, Marie Michael, Caroline Jan, Alberto Nassi, Sophie Lecourtois, Antoine Blancher, Emanuele Cozzi, Philippe Hantraye, Anselme L. Perrier

**Affiliations:** 1 CEA, DRF, Institute of Biology François Jacob, Molecular Imaging Research Center (MIRCen), 92265 Fontenay-aux-Roses, France; 2 CNRS, CEA, Paris-Sud Univ., Paris-Saclay Univ., Neurodegenerative Diseases Laboratory (UMR9199), 92265 Fontenay-aux-Roses, France; 3 CECS, I-STEM, AFM, Corbeil-Essonnes, 91100 France; 4 CORIT, Ospedale Giustinianeo, Padova, ITALY, Padova, Italy; 5 Transplantation Immunology Unit, Padua University Hospital, Padova, Italy; 6 Centre de Physiopathologie Toulouse-Purpan (CPTP), Université de Toulouse, CNRS, Inserm, Université Paul Sabatier (UPS), Toulouse, France; 7 Inserm U861, I-STEM, AFM, Corbeil-Essonnes, 91100 France; 8 UEVE U861, I-STEM, AFM, 91100 Corbeil-Essonnes, France

**Keywords:** Immune evasion, Huntington's disease, Induced pluripotent stem cells

## Abstract

Cell therapy products (CTP) derived from pluripotent stem cells (iPSCs) may constitute a renewable, specifically differentiated source of cells to potentially cure patients with neurodegenerative disorders. However, the immunogenicity of CTP remains a major issue for therapeutic approaches based on transplantation of non-autologous stem cell-derived neural grafts. Despite its considerable side-effects, long-term immunosuppression, appears indispensable to mitigate neuro-inflammation and prevent rejection of allogeneic CTP. Matching iPSC donors’ and patients’ HLA haplotypes has been proposed as a way to access CTP with enhanced immunological compatibility, ultimately reducing the need for immunosuppression. In the present work, we challenge this paradigm by grafting autologous, MHC-matched and mis-matched neuronal grafts in a primate model of Huntington’s disease. Unlike previous reports in unlesioned hosts, we show that in the absence of immunosuppression MHC matching alone is insufficient to grant long-term survival of neuronal grafts in the lesioned brain.

## Abstract (web summary)

Matching iPSC donors’ and patients’ HLA haplotypes has been proposed as a way to generate cell therapy products with enhanced immunological compatibility. Here the authors show that MHC matching alone is insufficient to grant long-term survival of neuronal grafts in the lesioned brain of non-human primates.

## Introduction

Clinical trials using fetal cells in Parkinson’s and Huntington’s diseases (HD) have paved the way for the development of human pluripotent stem cell (hPSC)-based replacement strategies in the brain. These pioneering trials have demonstrated frequent allo-immunisation to fetal donor antigens, sometimes associated with neuro-inflammation and rejection^1^. Despite the increased risk of cancer, infection and cardiovascular diseases, long-term immunosuppression is still used to protect allogeneic neural grafts from rejection^2,3^. Availability of induced-hPSCs (iPSCs) derived from the patient himself or from selected donors with some degree of HLA matching opens up opportunities to secure scalable sources of cell therapy products (CTP) with enhanced (e.g., HLA A, B, DR triple homozygous human iPSC or full immunological compatibility (e.g., autologous iPSC).

Some pre-clinical studies using autologous (AU) or syngeneic iPSC-derived grafts, the ideal immunological combinations, showed that such grafts can be well tolerated even in non-immune privileged sites in humanized mice and in the non-lesioned brain of non-human primates (NHPs)^4,5^. In contrast, others have reported that mouse and human iPSC derivatives can be immunogenic in syngeneic or AU recipients and in an AU humanized mouse model, respectively^6–8^. Interestingly, it has also been shown that a host immune response (T-cell infiltration) associated with necrosis following transplantation of syngeneic iPSC, appears to be dependent on the antigenic profile of the transplant^6,9^. Recently, the more economically sustainable “MHC-paradigm”, i.e., matching the major histocompatibility complex (MHC) of both donor and recipient^10^, was tested in NHPs using retinal^11^ or dopaminergic transplants^12^. Both studies showed no overt sign of humoral or cellular immune response directed against MHC-matched (MA) grafts and demonstrated improved engraftment of such transplants.

**Fig. 1:**
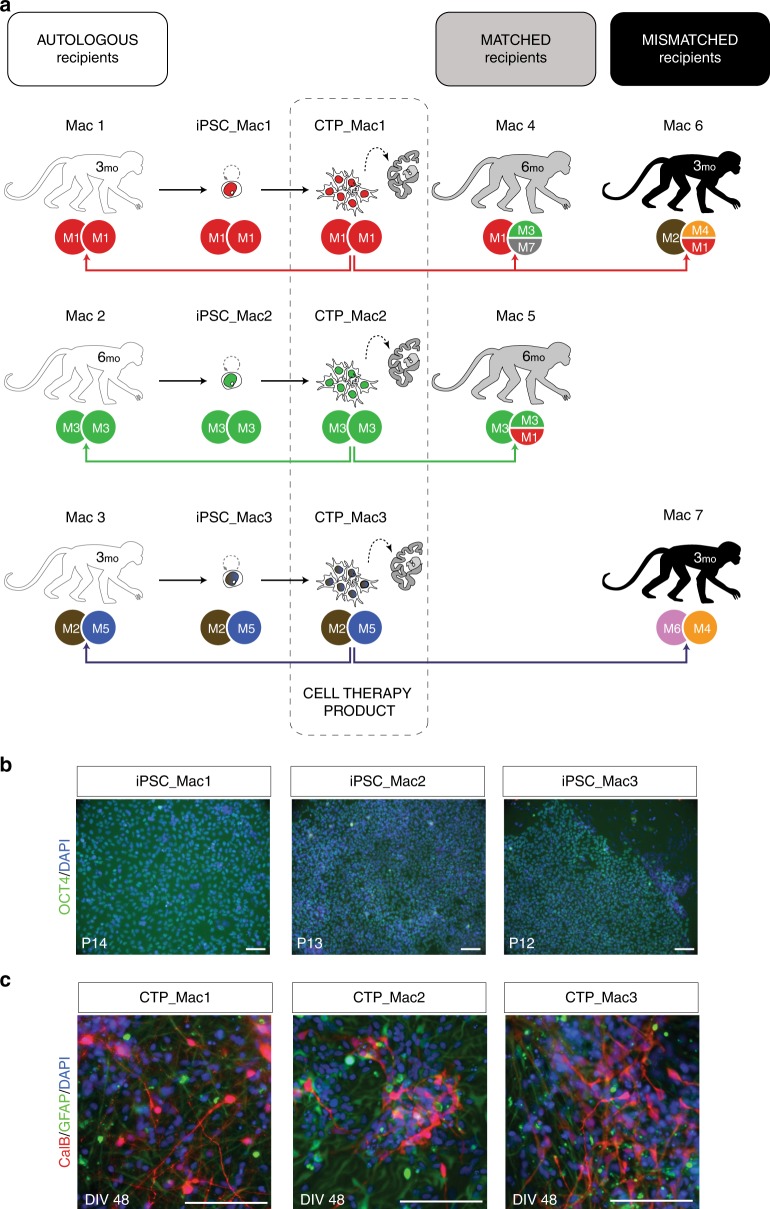
Generation and in vitro characterization of cell therapy products (CTPs) with specific MHC haplotypes. **a** Schematic representation of the experimental groups for intra-striatal cell transplantation in HD macaques (Mac) showing autologous (white, Mac 1–3), MHC-matched (gray, Mac 4–5) and MHC mismatched (black, Mac 6–7) recipients of CTPs derived from iPSC lines generated from PBMCs drawn from MHC homozygous (M1/M1; M3/M3) or heterozygous (M2/M5) cell donors (white). Survival time post-transplantation is indicated on each subject as 3 or 6 months (mo). **b** Immunohistochemistry results showing OCT4/DAPI double staining of each of the 3 iPSCs lines derived from PBMC NHP donors (Mac 1, 2, and 3) at passage (P) 14, 13, and 12 respectively. **c** Staining of CTPs derived from each of the iPSC batches at in vitro differentiation day (D.I.V.) 48 showing staining for Calbindin (CalB) and astrocytes (GFAP). Scale bar: 100 µm

Among all living *Macaca fascicularis* (*Mafa*) populations, that of Mauritius island presents the lowest genetic diversity because of its genetic isolation since its foundation^13,14^. In this study we take advantage of reduced genetic diversity in Mauritian macaques to challenge the “MHC-paradigm” by transplanting neuronal grafts in an excitotoxin-lesion model of HD in NHPs and by comparing the immunogenicity of AU, MA and fully mismatched (MI) grafts (Fig. 1a). The first step is to generate several iPSC lines with specific MHC haplotypes and the corresponding striatal neuronal grafts then to control their properties in vitro and in vivo in quinolinic acid-lesioned nude rats. The second step is to assess the immunogenicity of the three types of transplants in vivo in AU, MA, and MI NHP recipients at 3 and 6 months after transplantation in the lesioned brain, in the absence of peripheral immunosuppression. We observe a local infiltration of immune cells including CD8^+^ T cells in the core graft in allogenic MI and MA recipients that is significantly higher than that detected in AU recipients or in non-transplanted, QA-lesioned NHP. Our results suggest a delayed but ongoing sub-acute rejection in MA recipients. We thus conclude that MHC matching alone is insufficient to grant long-term survival of neuronal grafts in an excitotoxin-lesion model of HD in NHPs.

## Results

### Production of MHC-typed striatal grafts

**Fig. 2:**
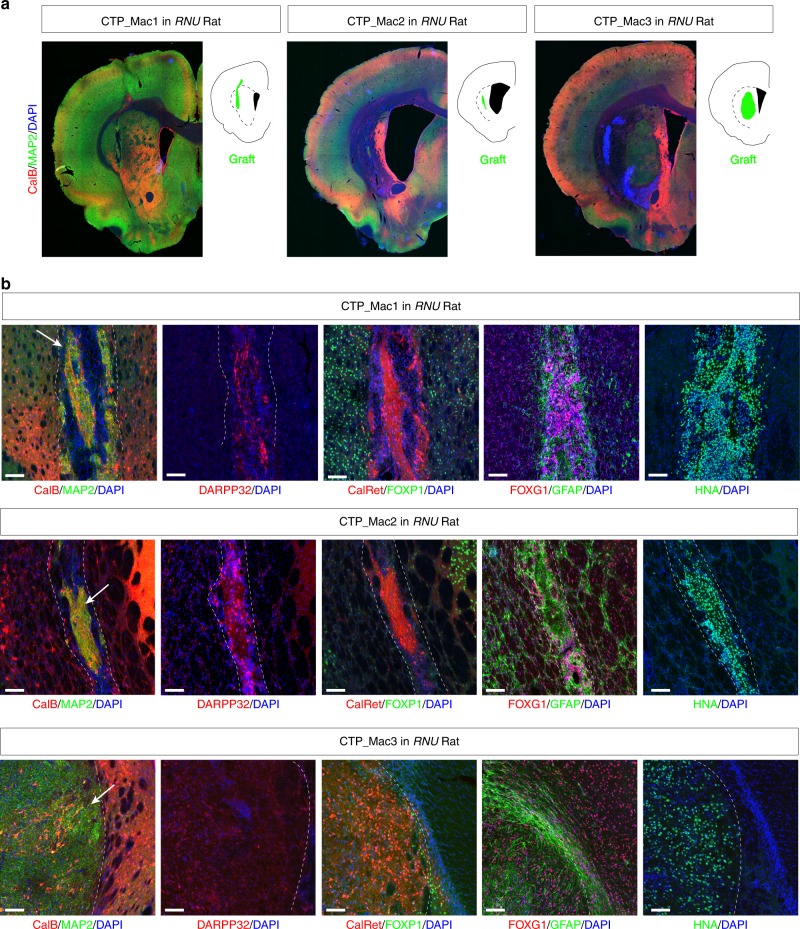
In vivo characterization of CTP batches derived from NHP iPSCs in quinolinic acid-lesioned nude rats (RNU). **a** Representative coronal slices showing the localization of the graft in the striatum and immunostaining with striatal markers at 10 weeks post-grafting (Calbindin, CalB; MAP2). **b** Immunohistochemistry results illustrating the composition of the graft (white dotted lines, white arrow) using striatal medium spiny neurons markers (CalB, DARPP32, FOXP1), striatal interneurons marker (Calretinin: CalRet), human and NHP cell nuclear marker (HNA) and the astrocyte marker GFAP. Scale bar: 200 µm

In order to generate MA striatal grafts, we genotyped NHP recipients and identified AU, MA or fully MI recipients with specific haplotypes. We identified two MHC homozygous individuals (M1/M1 and M3/M3, respectively) and derived two iPSC lines from peripheral blood mononuclear cells (PBMCs) (Fig. 1a). A third line, derived from PBMCs of a MHC heterozygous individual (M2/M5) was used as a control (Supplementary Table 1; Supplementary Fig. 1). All three lines expressed the pluripotency marker OCT4 (Fig. 1b) and were differentiated into striatal neuron precursors as previously described^15^. The differentiation potential into striatal neurons (Calbindin+) or glia (GFAP+) of each CTP batch was first controlled in vitro (Fig. 1c). The viability and in vivo differentiation potential into telencephalic neurons (FOXG1+, MAP2+) and more specifically, striatal projection neurons (Calbindin+, DARPP32+) or interneurons (CalRetinin+) of each batch of CTP transplanted in NHPs was also controlled in quinolinic acid-lesioned nude rats (Fig. 2).

**Fig. 3:**
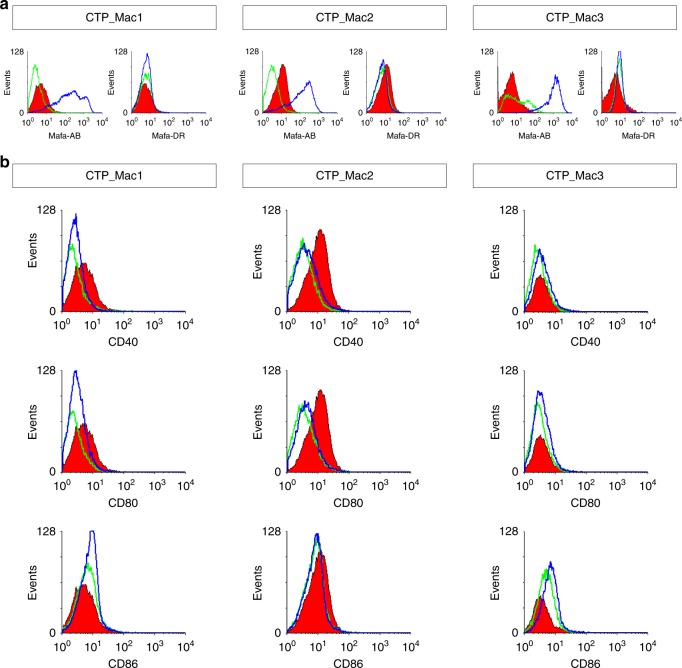
Expression of immune-related surface antigens on CTPs. **a** Representative histograms showing the expression of MHC molecules by each batch of CTPs (Mafa-A Mafa-B counterparts of HLA-A and HLA-B, respectively and Mafa-DR counterpart of HLA-DR) and **b** costimulatory molecules (CD40, CD80 and CD86) on CTPs under normal culture conditions (Untreated- green histograms) or after exposure to IFNγ (100 ng ml^−1^, blue histogram) for 48 h prior to analysis. The red histogram represents the isotype control

To assess the potential immunogenicity of CTPs at the time of grafting into immunocompetent NHPs, we measured the level of expression of MHC class I & II antigens, of T-cell co-stimulatory molecules, and of two key immunosuppressive cytokines, IL-10 and TGFb1, as previously described^7^ (Fig. 3 and Supplementary Fig. 2). Our results showed that CTPs weakly expressed MHC-I antigens (Mafa Class-I A, B measured by cross reacting anti-HLA-A, B, C antibodies) and did not express MHC-II antigens (Mafa Class-II DR measured by cross-reacting anti-HLA-DR antibodies) or any of the three major co-stimulatory proteins we analyzed (CD40, CD80 and CD86) (Fig. 3a, b). As expected, interferon gamma (IFN-γ) stimulation only increased MHC-I expression (Fig. 3a). Elevated levels of IL10 and TGFB1, that have been previously linked to tolerogenic immune response to autologous-iPSC derivatives in mice^7^, were not observed in Mac_iPSC-derived striatal CTPs (Supplementary Fig. 2).

### Graft localization, survival, and neuronal identity in NHPs

**Fig. 4:**
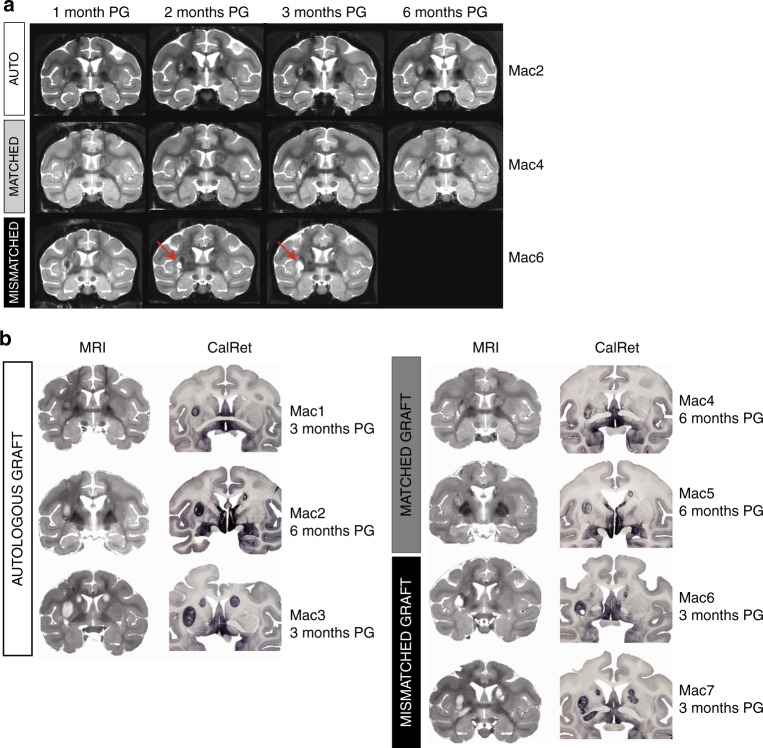
Graft size, location in QA-lesioned NHPs analyzed in vivo with MRI. **a** Representative coronal images of the longitudinal MRI follow-up of CTP derivatives after intra-striatal transplantation in autologous (white), MHC-matched (gray) and MHC mis-matched (black) recipients showing areas of hyper- (red arrows) and hypo-intensity in the caudate (bilateral) and putamen (unilateral, left) corresponding to the transplanted CTPs at 1, 2, 3 and, where applicable, 6 months post-surgery. **b** Graft MRI images prior to euthanasia and on post mortem sections stained with CalRet in autologous (white), MHC-matched (gray) and MHC mismatched (black) recipients at end-point (3 months PG for Mac 1, 3, 6, 7; 6 months PG for Mac 2, 4, 5)

**Fig. 5:**
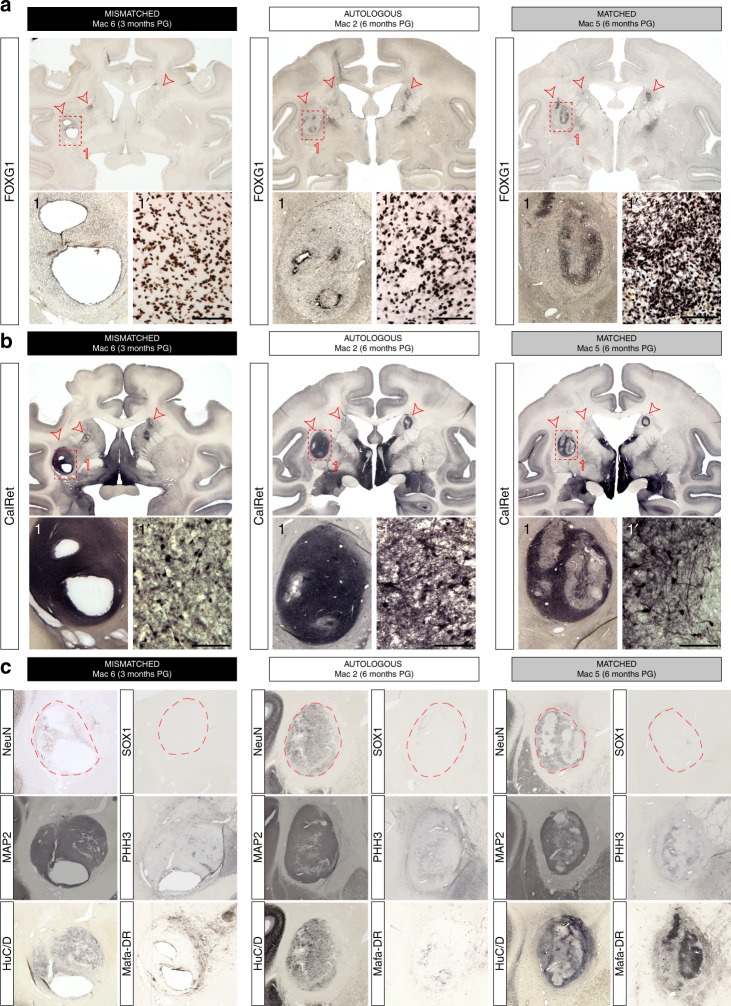
Histological assessment of graft localization, survival and neuronal identity of the grafts in mismatched (3 months PG), autologous (6 months PG) and matched (6 months PG) NHP recipients. **a**, **b** Immunohistological staining of coronal sections of the brain at the level of graft with anti-FOXG1 (**a**) and Calretinin (**b**) antibodies. Red arrows indicate the location of the grafted cells. **c** Brain slices were stained with post-mitotic neuronal markers NeuN (cell nuclei), MAP2 (soma and neuritic extensions), HuC/D (peri-nuclear soma), SOX1 (immature neural cells), and PHH3 (proliferative cells) compared to Mafa-DR (MHC II). Red dotted lines represent graft contour. Scale bar: 100 µm

Next, we assessed the immunogenicity of our three CTPs in vivo in AU, MA, and MI NHP recipients at 3 and/or 6 months after quinolinic acid-lesion and transplantation in the absence of immunosuppression. Group 1 (*n* = 4) consisted of paired AU and MI recipients analyzed at 3 months post-grafting (PG). Group 2 (1 AU and 2 MA recipients) was analyzed at 6 months PG (Fig. 1a). Group 3 consisted of non-transplanted QA-lesioned NHPs (*n* = 4). Magnetic resonance imaging (MRI) analysis revealed that QA lesions were detected only transiently (up to 1 month post-QA) as blurred-edged hyper-intensity signals on T2-weighted images^16^ and as dark hypo-intense regions at 3 and 6 months post-QA. CTPs were detected as sharp-edged hyper-intense areas from 2 months PG onwards (Fig. 4a). This allowed an accurate longitudinal follow-up of graft size and location that was ultimately validated by post mortem analysis (Fig. 4b). Fully MI recipients showed a variable intensity of MRI signals including hyper-intense liquid-filled cavities in the core graft detected early (2 months PG) (Figs. 4b and 5).

Histological analyses at 3 and 6 months PG confirmed the survival, location and telencephalic (FOXG1+), neuronal (NeuN+, MAP2+, HuC/D+) and striatal (Calretinin: CalRet+, Calbindin: CalB+) identity of graft-derived cells in all recipients (Fig. 5 and Supplementary Figs. 3–5). All grafts were DARPP32-negative (Supplementary Figs. 4 and 5). Large cavities were only detected in MI recipients (Fig. 5). The pattern of FOXG1+, CalRet+,and NeuN+ staining appeared more homogeneous in AU grafts and more heterogeneous in MA recipients both at 3 and 6 months PG (Fig. 5 and Supplementary Figs. 4 and 5). The proliferation potential of the different grafts assessed with the Phospho Histone H3 (PHH3) antibody showed that the number of actively dividing cells at 3 months was greater than that observed at 6 months PG regardless of the donor-recipient combination (*T*-test, *p* = 0.02), and that their density was always inferior to 1% of the graft (Fig. 5 and Supplementary Fig. 3).

### Infiltration of immune cells in MI and matched grafts

**Fig. 6:**
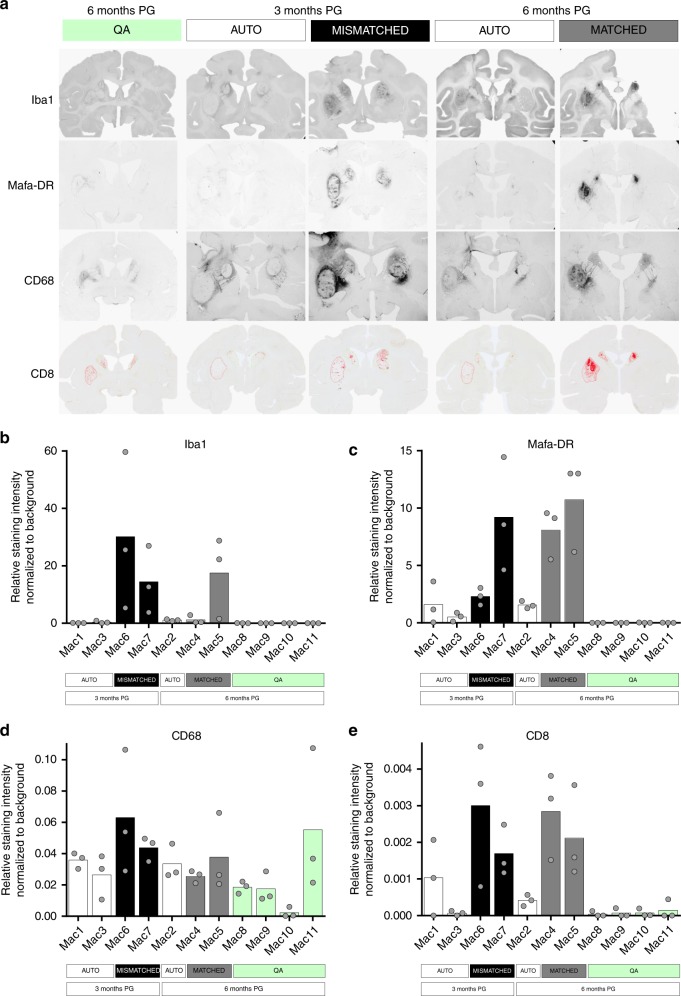
Post mortem analysis of the immune markers in QA-lesioned NHPs after CTP transplantation. **a** Representative coronal sections of the NHP brain at the level of the graft (commissural) showing staining for microglia/macrophages (Iba1; CD68), MHC Class II positive cells (revealed by an anti HLA-DR antibody cross-reacting with Mafa-DR antigens), and cytotoxic T-cells (CD8, cartography,) in quinolinic acid lesioned (QA) untransplanted controls (green) and in autologous (AU, white), MHC-matched (MA, gray), and mismatched (MI, black) CTP recipients at 3 or 6 months post-grafting (PG). **b**–**e** Quantification of Iba1 (**b**), Mafa-DR (**c**), CD68 (**d**) and CD8 (**e**) immunoreactivity in AU, MA, MI CTP recipients at 3 or 6 months post-grafting (PG) and in untransplanted controls. Bar graphs represent mean value of the three regions of interest considered (left and right caudate, and left putamen) for each animal; gray dots represent individual values for each region (*n* = 3 biologically independent cell deposit per animal)

Post mortem analysis at 6 months post-QA or PG revealed the presence of host-derived immune cells expressing Iba-1, MHC-class II (revealed by anti-HLA-DR monoclonal antibodies), CD68, CD45, CD8 and CD4 (Fig. 6 and Supplementary Figs. 4–6). Non-transplanted QA-lesioned controls presented an activation of macrophages (CD68 + ) and little to no activation of infiltrating immune cells (CD45+, CD8+, CD4+)^17^. In this context, AU grafts elicited little to no additional activation of these infiltrating immune cells. In contrast, MI recipients presented a considerable infiltration and marked staining for Iba1, MHC-class II, CD68, CD8, and CD4 around the graft and cavities (Fig. 6 and Supplementary Fig. 6b). These findings suggest an ongoing rejection process of the allogeneic graft in MI recipients and are in agreement with clinical observations of allo-immunisation leading to rejection in HD patients transplanted with fetal grafts^1^. More interestingly, the staining in MA recipients at 6 months PG was closer to that of MI recipients at 3 months PG than to the staining of AU grafts at 3 and 6 months PG. Accordingly, a clear infiltration and a strong staining for most markers (Iba1, MHC-class II, CD45, CD8, CD4) could be observed, suggesting a delayed but ongoing sub-acute rejection.

### Absence of humoral response to  autologous and allogenic grafts

**Fig. 7:**
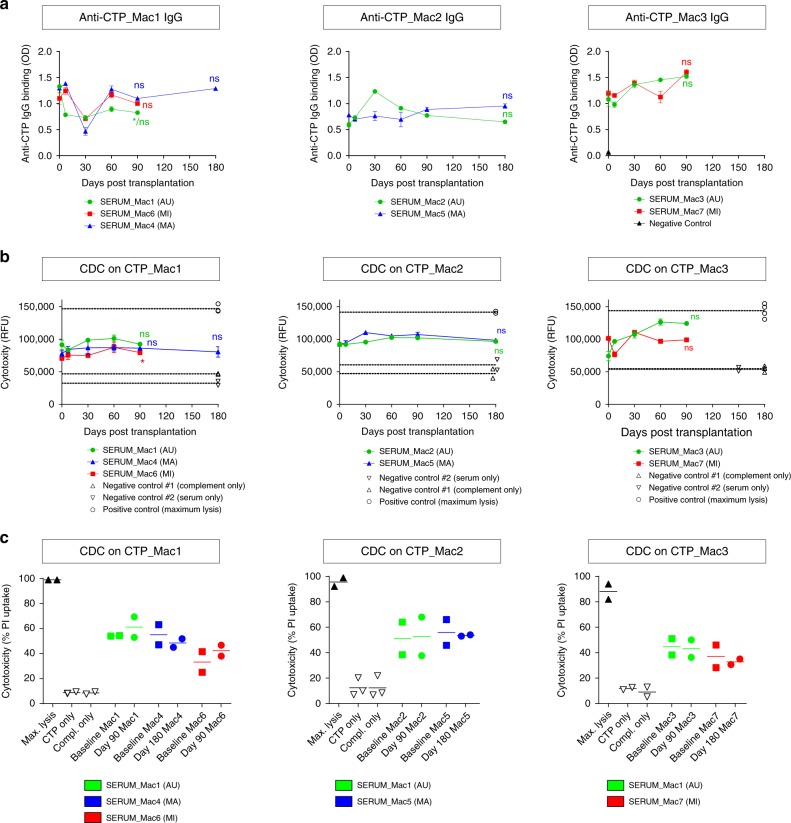
Detection of hemolytic CTP-specific antibodies in the serum of transplanted macaques. The sera of AU, MA, MI recipients were collected before and at different time-points following transplantation. **a** The presence of anti-CTP IgG was detected by cell-based ELISA. **b** The capability of anti-graft antibodies to trigger the complement-dependent cytotoxicity was also evaluated by measuring the fluorescence signal of the Cell-Tox Green probe (for (**a**) and (**b**) data are expressed as mean ± s.e.m of *n* = 3 replicates, one-way ANOVA followed by Dunnett’s multiple comparisons post hoc test using day 0 as control; for **b** positive and negative controls: individual replicates shown) or **c** by measuring the percentage of the dead cells (propidium iodide-positive cells) by flow cytometry (*n* = 2 technical replicate of single biological samples, mean per time point)

Finally, the humoral response against all three types of grafts and their ability to trigger complement-dependent cytotoxicity (CDC) was assessed in vitro monthly. Unexpectedly, before transplantation or QA lesion, pre-existing anti-graft antibodies were observed even in the context of AU combinations, and such antibodies had a cytotoxic activity primarily mediated by IgM. However, no transplant combination elicited a significant increase in the serum levels of anti-graft antibodies or CDC activity at any time-point following transplantation (Fig. 7 and Supplementary Fig. 7). Unlike a recent report using allogeneic retinal transplants in NHPs^18^, our data shows the absence of transplant-elicited humoral immune response against the three CTPs used in this study. On the other hand, a longitudinal measurement of six cytokines in the serum and CSF did not reveal a cytokine profile compatible with a T cell-based rejection of the iPSC-derived grafts (Supplementary Fig. 8).

## Discussion

Our results suggest that, unlike AU neuronal grafts, allogenic (MI and MA) grafts elicit a local infiltration of CD8^+^ T cells and an increase in local HLA-DR staining, far beyond the effect observed in the QA-lesioned non-transplanted NHPs. In our NHP model of HD, in the absence of immunosuppression, haplotype-matching was insufficient to grant long-term survival of neuronal grafts and appeared to trigger a delayed/attenuated immune response in the host. Our results are not in agreement with the main claim of Morizane and colleagues^12^ who showed that, in the absence of immunosuppression, MHC matching improved engraftment of iPSC-derived TH+ neurons in the non-lesioned brain of NHPs and reduced the immune response by suppressing the accumulation of microglia (Iba-1+) and lymphocytes (CD45+) into the grafts. It is noteworthy that the extent of MHC matching in both studies are similar. At least four hypotheses may explain the discrepancies between our findings and those previously published. Firstly, transplantation into intact versus lesioned animals might not yield the same results in terms of cell maturation and/or rejection. Indeed, even though acute excitotoxic lesions may bear only limited resemblance to the chronic and progressive inflammation observed in HD patients, transplanting into a lesioned brain may be more pertinent as it more closely mimics the inflammatory milieu associated with neurodegenerative disorders in man. Secondly, the shorter duration of the follow-up in the dopaminergic neuronal MA grafts study may have affected the timely identification of an ongoing immune process. Thirdly, the antigenic profile, closely related to the maturation time indispensable to generate fully differentiated striatal iPSC-derived neurons, may substantially differ from that of dopaminergic neurons. This may ultimately result in a different, possibly more aggressive, anti-graft immune response. In all cases, our post mortem data suggest that grafts are largely composed of post-mitotic striatal neurons and that maturation is time-dependent rather than dependent on the recipient-donor MHC matching combination (AU, MA, MI). Finally, the extent of cell division and/or the presence of immature cells may give rise to areas with immature or leaky BBB and thus be more prone to trigger the host’s immune response.

In conclusion, in the specific context of brain transplantation, our pre-clinical data suggest that a better immunological match is not necessarily associated with significantly improved survival and maturation of the grafts. Alternative strategies allowing CTPs to escape altogether the host cell-mediated immune response^19,20^ or a combination of MHC-matching and peripheral immunosuppression should be further investigated in NHPs to allow stem cell therapy to achieve its full therapeutic potential.

## Methods

### NHPs: ethics and housing

All animal studies were conducted according to European regulations (EU Directive 2010/63) and in compliance with Standards for Humane Care and Use of Laboratory Animals of the Office of Laboratory Animal Welfare (OLAW—no. #A5826–01) in a facility authorized by local authorities (authorization no. #B92-032-02). The experimental protocol was reviewed and approved (authorization no.14_019) by the local ethics committee (CETEA No.44). All efforts were made to minimize animal suffering and animal care was supervised by veterinarians and animal technicians skilled in the healthcare and housing of NHPs. All primates were housed under standard environmental conditions (12-h light-dark cycle, temperature: 22 ± 1 °C and humidity: 50%) with ad libitum access to food and water. Experiments were conducted on a total of 11 male cynomolgus monkeys (*Macaca fascicularis*, supplied by Noveprim, Mauritius Island) of a mean age of 5.4 ± 0.2 years and a mean weight of 5.4 ± 0.3 kg.

### Genotyping of NHPs

Genomic DNA for all NHP considered was extracted from peripheral blood sample using QIA amp Blood Kit (QIAGEN, Courtaboeuf, France). The animal MHC genotypes were determined with by means of genotyping 17 microsatellites scattered across the MHC region^14^. Genotypes were determined with DNA Size Standard-kit- 600 (Beckman Coulter, Villepinte, France) after denaturation and separation of the amplification products by capillary electrophoresis with a CEQ8000 analyzer and scored with the software CEQ8000 Genetic Analysis System v8.0 (Beckman Coulter, Villepinte, France). Primer used to amplify the 18 MHC microsatellites were the following: D6S2972 AAATGTGAGAATAAAGGAGA and GATAAAGGGGAACTACTACA; D6S2970 TCCCATGGTCAAGTTCTCAG and TCATGGATCTTATCAGCCTC; D6S2854 TCATGAGCGTGGCACTGCAC and CCGTATGTTGCAACCAGGAG; D6S2704 TTTTGCCACTCTGGAGGATGGG and GAGCATAATATCTGGTCTACTGC; D6S2847 TATTGGACAGCACTGCTCTGG and TGCCATTCAGATTGGTTTTTCTG; C4-2-25 ATGTTAGTTTTAGAAGATAACACTC and TCTTCTGTGCAAGCAAGCACTGTAC; D6S2691 GTAGCTGTGGAAACAGTGTCCATG and CTTGACTTGAAACTCAGAGACC; MICA CCTTTTTTTCAGGGAAAGTGC and CCTTACCATCTCCAGAAACTGC; D6S2793 CTACCTCCTTGCCAAACTTGCTATTTGT and AATAGCCATGAGAAGCTATGTGGGGGA; D6S2782 TTTACTTGCTCTCACTCTCAGGCC and GGAAGACATTAACTTGTTAGCA; D6S2669 TGCCTTCCGTAAGCCTCAGTCT and TTAAGGACAGCAAAGCCAGCAGCA; D6S2892 TGCATGTCCTGTGAGGTAAG and ACTCAACCCTGCTGTTGTAG; DRACA TGGAATCTCATCAAGGTCAG and ACATTTGTATGCTTCAGATG 2 D6S2876 GGTAAAATTCCTGACTGGCC and GACAGCTCTTCTTAACCTGC; D6S2747 AGGAATCTAGTGCTCTCTCC and CTCTAGCAAAAGGAAGAGCC; D6S2745 CCTAGAGATTCCTCCACATTA and CCAATGTTTGATAGCAGACTGGGGT; D6S2741 AGACTAGATGTAGGGCTAGC and CTGCACTTGGCTATCTCAAC; D6S2771 ATTCCTTTCACTAGTTCTGG and CCACTTTAAGAAATTAGAAAAG from refs. ^14,21,22^

From the genotypes obtained for the 17 microsatellites, the most probable haplotypes were deduced for each animal^14^. As reported previously, seven founder MHC haplotypes (M1 to M7) were identified in the Mauritian macaque population as well as recombinant haplotypes derived from these seven haplotypes^14,23^. The genotypes of the animals are shown in Supplementary Fig. 1 and Table 1.

### Imaging in NHPs

Magnetic resonance imaging (MRI) was performed on all NHPs at baseline in order to determine the stereotactic coordinates for surgery and was repeated monthly to monitor grafts.

Primates were induced with ketamine (1 mg kg^−1^) and xylazine (0.5 mg kg^−1^), maintained anesthetized with an intravenous (i.v.) infusion of propofol (1 ml kg^−1^ h^−1^) and placed in the magnet in a sphinx position with the head fixed in a stereotactic MRI-compatible frame (M2E, France). NHPs were heated by a hot air flux and their temperature and respiration parameters monitored remotely.

All acquisitions were performed on a horizontal 7 T Varian scanner (Palo Alto, CA, USA) equipped with a gradient coil reaching 100 mT m^−1^ (300 µs rise time). A surface coil (RAPID Biomedical GmbH, Rimpar, Germany) was used for transmission and reception. T2-weighted images were acquired using a high-resolution 2D fast spin-echo sequence (469 × 469 µm² in-plane resolution, 1 mm slice thickness, 70 slices), with echo time TE/ Repetition time TR = 20/8000 ms, 5 echoes, effective TE = 52.5 ms and acquisition time Tacq = 43 min. For T2*-weighted images the parameters used were: 469 × 469 µm² in-plane resolution, 1 mm slice thickness, 40 slices, 5 TE (from 5.5 to 30 ms), repetition time TR = 2 ms and acquisition time Tacq = 8 min.

### Surgery in NHPs

Primates were induced with ketamine (1 mg kg^−1^) and xylazine (0.5 mg kg^−1^), and maintained anaesthetized with an i.v. infusion of propofol (1 mg kg^−1^ h^−1^) with constant monitoring of blood pressure, heart rate, exhaled CO_2_, temperature, and respiratory frequency. NHPs were placed in a stereotactic MRI-compatible frame in a sphinx position with the head resting on a mouth bar and fixed by ear bars.

An intradermal injection of local analgesics (Bupivicaine (1 mg kg^−1^), xylazine with adrenaline (2 mg kg^−1^)) was administered before incision of the skin and before suturing the cutaneous plane. A wide spectrum antibiotic was delivered before and after surgery (TLA terramycin long action, 20 mg kg^−1^).

All NHPs received two bilateral pre-commissural caudate and two unilateral post-commissural left putamen injections of 80 mMol quinolinic acid (QA) prepared dissolving QA in 1 N NaOH and subsequently diluting in phosphate buffer saline heated at 50 °C (pH was adjusted between 7 and 7.4). At each coordinate, a single deposit of 10 µl of 80 mM QA toxin was injected at a rate of 1 µl min^−1^ using a Hamilton syringe and 26 G needle controlled by a KDS injection micropump (Phymep, France). Two weeks following QA lesion, primates in groups 1 and 2 were transplanted with either CTP_Mac1, CTP_Mac2, CTP_Mac3. 3.375 M cells were delivered into the left putamen (3 tracks, 2 × 5 µL deposits track^−1^ 2 mm apart D/V): 1 track 1 mm anterior of the most anterior QA lesion site in the putamen, 1 track equidistant of the two lesion sites in the putamen and 1 track 1 mm posterior of the second lesion site in the putamen). 1.125 M cells were delivered as 3 into the left and right caudate nucleus (3 tracks, 1 × 5 µL deposit track^−1^): 1 track 1 mm anterior of the first QA lesion site in the caudate nuclei, 1 track equidistant of the two lesion sites in the caudate nuclei and 1 track 1.5 mm posterior to the second lesion site in each caudate nucleus.

### Post mortem analysis in NHPs

Three or six months post-transplantation, NHPs were induced with ketamine (1 mg kg^−1^) and xylazine (0.5 mg kg^−1^) before receiving an i.v. lethal dose of pentobarbital (100 mg kg^−1^, Sanofi, France). NHPs were then transcardially perfused with 0.9% NaCl followed by 4% paraformaldehyde and the brains were extracted and cryoprotected in incrementing PBS-sucrose gradients before histological processing. Post-fixed brains were sectioned coronally with a freezing stage microtome (Leica SM2400) into 40 µm thick sections. Free floating tissue sections were incubated for 48 h at 4 °C with the following primary antibodies: NeuN 1:5000 (MAB377, Millipore, Billerica, MA); FOXG1 1:500 (AB18259, Abcam, Cambridge, UK), Iba1 1:1000 (1919741,Wako, Osaka, Japan), CD8 1:100 (A07757, Beckman Coulter, Villepinte, France.), CD68 1:5000 (M0718, DakoCytomation, Glostrup, Denmark), CD4 1:800 (M7310, DakoCytomation, Glostrup, Denmark), CD45 1:1000 (M0701, DakoCytomation, Glostrup, Denmark), Calretinin 1:5000 (AB5054, millipore, Billerica, MA), Calbindin1:1000 (C9848, Sigma, st Louis, MO, US), PHH3 1:1000 (06-570, Millipore, Billerica, MA), GFAP 1:5000 (Z0334, DakoCytomation, Glostrup, Denmark), MAP2 1:800 (M1406, Sigma), FoxP1 1:800 (Ab32010, AbCam, Cambridge, UK), HNA 1:400 (mab1281, Millipore, Billerica, MA), OCT3/4 1:200 (sc5279, Santa Cruz, Dallas, Texas), Sox1 1:200 (4194, Cell signaling, Danver, MA), HuC/HuD 1:500 (A-2127, ThermoFisher, Waltham, MA), DARPP-32 1:100 (2302 Cell signaling, Leiden, Netherlands), anti-HLA-DR 1:500 (MO746, DakoCytomation, Glostrup, Denmark; this antibody cross reacts with Mafa-DR antigens, the cynomolgus macaque counterpart of HLA-DR antigens), diluted in PBS, 0.2% triton, and 3% normal goat serum solution. After washing, sections were incubated for 1 h at room temperature with the appropriate biotinylated secondary antibodies (anti-rabbit, anti-mouse, 1:1000 Vector laboratories) and the avidin biotinylated enzyme complex (Vectorstain ABC kit) applied and visualized using diaminobenzidine. Post mortem histological analysis included qualitative morphological analysis at different magnifications and quantitative measurements were performed using a computer assisted analysis system consisting of a Leica DM6000 microscope and Mercator software (ExploraNova, La Rochelle, France). For inflammatory reaction measurements, outlines of the striatum were first delineated using Mercator software and then the inflammatory area defined using GFAP or Mafa–DR staining threshold was extracted from the background. For PHH3-positive cells, Alexa-594 secondary antibody (anti-rabbit, 1:500, Invitrogen) was used with DAPI counter staining of nuclei. PHH3-positive cells were manually counted in the entire graft area, DAPI-positive nuclei were counted in randomly selected sub-regions of the core graft. For CD8-positive cells cartographies, sections were first scanned using Axioscan Z1 slides scanner (Zeiss, Oberkochen, Germany), positive-cells were then extracted from the background using staining threshold.

### *Macaca fascicularis* iPSC derivation

Cynomolgus PBMCs were extracted from blood sample and reprogrammed into induced pluripotent stem cells (iPSCs) using the CytoTune®-iPS 2.0 Sendai Reprogramming Kit (Life Technologies). Ten iPSC clones were expanded on MEF in DMEM/F12 Glutamax supplemented with 20% PluriQ serum replacement (AMSBIO), 1 mM nonessential amino acids, 0.55 mM 2-mercaptoethanol, and 10 ng ml^−1^ recombinant human FGF2 (Miltenyi). Cultures are fed daily and passage with collagenase IV every 3–4 days. Clones were banked and their pluripotency was assessed using alkaline phosphatase and OCT4 staining. We performed RT-PCR analysis on Mac1,2,3_iPSC lines to detect the SeV genome and transgenes according to the Invitrogen’s user guide. We performed end-point RT-PCR with four pairs of primers either targeting all three sendai viruses used during reprogramming (*SeV*: GGA TCA CTA GGT GAT ATC GAG C and ACC AGA CAA GAG TTT AAG AGA TAT GTA TC target amplicon = 181 bp) or targeting each vector individually (*KOS*: ATG CAC CGC TAC GAC GTG AGC GC and ACC TTG ACA ATC CTG ATG TGG (528 bp) for CytoTune™ 2.0 KOS; *c-Myc*: TAA CTG ACT AGC AGG CTT GTC G and TCC ACA TAC AGT CCT GGA TGA TGA TG (532pb) and *Klf4*: TTC CTG CAT GCC AGA GGA GCC C and AAT GTA TCG AAG GTG CTC AA (410 bp) for CytoTune™ 2.0 hc-Myc, and Klf4. We used human fibroblasts, 7 days post exposure to those three sendai-viruses as positive control. No specific amplification at the expected size (Supplementary Fig. 10) was detected in any of these analyses for Mac1, Mac2, and Mac3_iPSC cells.

### iPSC differentiation (production of CTPs)

Striatal neuron progenitors were derived from NHP-iPSC as described for human ESC/iPSC^15^. NHP-iPSC clones are manually detached from MEFs and aggregated for 6 h in neural induction medium consisting of DMEM/F12, neurobasal, N2 and B27 supplement, β-mercaptoethanol and Penicillin/Streptomycin (N2B27 medium) supplemented with 0.1 µM LDN-193189 (Sigma Aldrich), 20 µM SB-431542 (Tocris), 1 µM XAV-939 (Tocris) and 10 µM ROCK inhibitor (Y27632, Miltenyi). The medium was replaced daily during the entire course of the differentiation. Supplementation with cytokines or drug was adjusted as follows: at day 1, NHP-iPSC-aggregates are transferred on poly-ornithin laminin-coated dishes without Y27632. At day 4, SB-431542 is removed from the medium. At day 7, LDN-193189 is removed and cells are alternatively treated, every other two-days, with XAV-939 and 50 ng ml^−1^ Sonic Hedgehog (464-SH R&D system), or SHH supplemented N2B27 medium, until day 15. From day 15 to 17, 18 or 19 days differentiation, cells are exposed to 0.5 mM dbcAMP (Sigma-Aldrich) and 0.5 mM valpromide (Lancaster Synthesis).

CTP cells produced from the corresponding iPSC lines after 17 to 19 days of striatal differentiation in vitro were washed once in PBS then collected using accutase (Invitrogen). CTPs were either resuspended at 75,000 cells µl^−1^ in HBSS supplemented with 0.05% DNase I or frozen down for further in vivo QC in rodent and in vitro QC analyses, in CryoStor® CS10 cryopreservation medium at a concentration of 0.5–3 × 10^6^ cells ml^−1^) (# 07930, STEMCELL Technologies) in a CRYOMED FREEZER (Thermo scientific). Freezing rate was 1 °C min^−1^ form +4 °C to −40 °C then 10 °C min^−1^ from −40 °C to −90 °C. Vials were then directly transferred at −135 °C into a liquid nitrogen vapor tank for long-term storage.

### In vitro CTP QC

For the in vitro QC of the CTP, frozen vials of CTP are thawed at 37 °C, washed once in N2B27 media then plated on poly-ornithin laminin-coated dishes at 100,000 cells cm^−2^ in terminal specification medium consisting of N2B27 media supplemented with 20 ng ml^−1^ BDNF (R&D Systems), 0.5 mM dbcAMP, 0.5 mM valpromide and 25 ng mL^−1^ Activin A (120–14, Peprotech). Analyses of partially or terminally differentiated CTP-derivatives are performed 1, 5 and 25 to 30 days in vitro after replating. Cells were fixed with 4% PFA for 10 min at room temperature and incubated with primary antibodies overnight at 4 °C. Secondary antibodies and DAPI counterstain were applied for 1 h at room temperature. The primary antibodies used were anti-Calbindin (rabbit, Swant), anti-GFAP (chicken, Millipore). Total RNA was isolated using the RNeasy Mini extraction kit (Qiagen) according to the manufacturer’s protocol. A DNase I digestion was performed to avoid genomic DNA amplification. RNA levels and quality were checked using the NanoDrop technology. A total of 500 ng of RNA was used for reverse transcription using the SuperScript III reverse transcription kit (Invitrogen). Quantitative polymerase chain reaction (qPCR) analysis was performed using a QuantStudio 12 K Flex real-time PCR system (Applied biosystem) and Luminaris Probe qPCR Master Mix (Thermo Scientific), following the manufacturer’s instructions. Gene expression was normalized to beta-Actin expression. Primer sequences for Macaca Fascicularis genes were: *ACTB*: 5′-CGCCATGGATGATGATATCGC-3′ and 5′-TGAATCCTTCTGACCCATGCC-3′. *TGFB1*: 5′- GGAAATTGAGGGCTTTCGCC-3′ and 5′- CCGGTAGTGAACCCGTTGAT-3′. *IL10*: 5′- CTGAGAACCACGACCCAGAC-3′ and 5′- GAAGAAATCGATGACAGCGCC-3′. *NANOG*: 5′- GGCGACTCACTTTATCCTGACT-3′ and 5′- CAAGCTTTGGGGACAAGCTG-3′. *DLX2*: 5′- GCACATGGGTTCCTACCAGT-3′ and 5′- TCCTTCTCAGGCTCGTTGTT-3′. Data are expressed as mean values ± s.e.m.

### Surgery in nude rats

All experimental procedures with rodents were performed in strict accordance with the recommendations of the European Commission (86/609/EEC) concerning the care and use of laboratory animals. Adult nude rats (weight 220–260 g at the time of grafting; Charles River Laboratories, Wilmington, MA, www.criver.com) were used. All surgical procedures were performed under full anesthesia using a mixture of ketamine (15 mg kg^−1^) and xylazine (3 mg kg^−1^; Bayer Healthcare, www.bayerhealthcare.com) and using a stereotaxic frame. Unilateral lesions were made by injecting 2 µl of 80 nmol µl^−1^ QA (1 µl per track) dissolved in 0.1 M PBS into the right striatum, according to the following coordinates (in millimeters): Track#1 anteroposterior (A) −0.4; lateral (L) −3.7; ventral (V) −4.0; Track#2 (A) + 1.2 (L) −2.9 (V)−4.0 (tooth bar −2.3). One week after the lesion, rats received transplants of 150,000 or 300,000 cells from frozen batches: CTP_Mac1, CTP_Mac2 or CTP_Mac3. CTP were thawed at 37 °C, immediately washed once in PBS then resuspended at 75,000 cells µl^−1^ in HBSS supplemented with 0.05% DNaseI. Lesioned rats were transplanted with 4 µl of CTP suspension (2 deposit of 2 µl per track, 1 track per striatum) according to the following coordinates (in millimeters): anteroposterior (A) + 0.4; lateral (L) −3.3; ventral (V) −4.0 and −5.0; and tooth bar −2.3. Ten weeks after transplantation rats were terminally anesthetized with 1 g kg^−1^ intraperitoneally sodium pentobarbital (Ceva Sante Animale, www.ceva.com), and their brain was fixed by transcardial perfusion with 100–150 ml of 0.1 M PBS (pH 7.4), followed by 250 ml of buffered 4% PFA. Brains were removed, postfixed overnight at 4 °C in 4% PFA, and then cryoprotected in 30% sucrose solution at 4 °C. Coronal brain sections (30 µm) were cut on a freezing microtome, collected serially (interspace, 480 µm), and stored at −20 °C in a cryoprotectant solution until analysis. For fluorescent immunohistochemical analyses, the brain sections were incubated with primary antibodies for 12 h at 4 °C. Secondary antibodies and DAPI counterstain were applied for 3 h at room temperature. Tile imaging of brain section were acquired on a ZEISS LSM880 confocal microscope with Zen Black acquisition software.

### Immunophenotyping of CTPs

The CTP lines examined in these studies were cultured in N2B27 media on poly-ornithin laminin-coated dishes at 100,000 cells cm^−2^. Culture medium with or without INF-γ (100 ng ml^−1^) was added for 48 h prior to cell harvesting with Accutase (StemPro Accutase, Thermo Scientific, Waltham, MA USA). Cell-surface markers were analyzed by flow cytometry using antibodies targeting human epitopes that are shared with *Macaca fascicularis (Mafa)*. The monoclonal antibodies used were directed against the following specificities: the anti-HLA-A, -B, -C clone G46-2.6, (BD-Pharmingen, San Diego, CA) cross reacts with Mafa-A and Mafa-B MHC class I antigens; the anti-HLA-DP, -DQ, -DR clone I3,9–49 (BeckmanCoulter Life Sciences, Milan, Italy) cross reacts with Mafa DR antigens; the anti-CD56 (NCAM) (clone MEM-188) (Biolegend, San Diego, CA, USA); the anti-CD40 (clone 5C3), anti-CD80 (clone L307.4), anti-CD86 (clone FUN-1) (all from BD-Pharmingen). Isotype-matched immunoglobulins were used as controls. Briefly, 1 × 10^5^ cells were incubated with PE-labeled antibodies for 30 min at 4 °C, washed with phosphate-buffered saline (PBS), 0.5% bovine serum albumin (BSA), and 0.1% Na-Azide. Flow cytometric analysis was performed on a FACScalibur flow cytometer (Becton Dickinson, San Jose, CA) using the CELLQuest program (Becton Dickinson). The data were analyzed by plotting forward scatter (FSC) versus side scatter (SSC) and by defining a R1 region. Control histograms (isotype controls) were overlaid onto the stained positive dataset allowing positive cells to be accurately identified on single parameter histograms (PE-FL2 or FITC-FL1 fluorescence). The lower limit of markers for the positive population were set to include less than 2% of events in the isotypic control (negative control). This gating strategy is illustrated in Supplementary Fig. 10a.

### Anti-graft antibody response

To assess the extent of the antibody response induced by CTP grafts, serum was obtained from each recipient prior to and at 7, 30, 60, 90, 180 days following implantation. Donor CTP were seeded into Poly-L-ornithine/Laminine coated 96-well ELISA plates (Polysorp, NUNC^TM^ Thermo Scientific, Waltham, MA USA). Briefly, 32.000 cells per well were incubated at 37 °C in 5% CO_2_ for 48 h, washed twice with PBS and fixed 30 min with 2% formalin (Sigma Aldrich). Plates were blocked overnight at 4 °C with PBS-5% BSA. In parallel, cell-free background plates were prepared and treated similarly. After washing twice with PBS-0.1% Tween, heat-inactivated (HI) recipient sera (in triplicate) were added at a dilution of 1:100, incubated at 4 °C for 1 h. Following four washes with PBS-0.1% Tween, horseradish peroxidase-conjugated goat anti-human Fc IgG immunoglobulins (Jackson ImmunoResearch Laboratories, West Grove, PA, USA) were added to each well and incubated at room temperature for 1 h. After washing four times, tetramethyl benzidine (TMB) solution (Scytec, West Logan, UT, USA), was added to each well. Reaction was blocked by adding a stop solution (H_2_SO_4_ 1 M, Sigma Aldrich, St. Louis, MO USA) and the plates were analyzed at 450 nm with an ELISA reader (Bio-Rad, Hercules, CA, USA). The absorbance value was calculated as the average value of the triplicate determination.

The anti-graft antibody response was further confirmed by flow cytometry. Briefly, cultured donor CTPs were detached and incubated with HI- recipient sera for 30 min at 37 °C. After washing with PBS-BSA-sodium azide, antibody binding was revealed using FITC-labeled anti-human IgG (Jackson ImmunoResearch Laboratories) or IgM (Dako, Milan, Italy) antibodies. Secondary antibody without serum incubation was assessed as a negative control. Median fluorescence intensity (MFI) was recorded by FACScalibur cytometer (BD Bioscience) acquisition and analyzed by CELLQUEST software (BD Bioscience).

### Complement-dependent cytotoxicity (CDC)

Donor CTPs were seeded into 96-well flat plates at a cell density of 32.000 cells well^–^^1^ in 100 µl of complete medium. After for 48 h, the supernatant was discarded and cells were incubated for 30 min at 37 °C with 50 µl of 1:4 diluted HI-sera collected from each recipient at baseline and following CTP transplantation. Negative control wells were incubated with culture medium without any serum. After incubation, supernatants were discarded and cells were further incubated with 150 µl of baby rabbit complement (Cederlane, Burlinghton, Ontario, Canada) diluted 1:10 at 37 °C for 1 h. Negative control wells received complement only or HI-serum without the addition of complement. Positive control wells consisted of cells exposed to a lytic solution. CellTox™ Green Dye (Promega, Madison, WI, USA) was added at the end of complement exposure and fluorescence (560em/590ex, Victor3^TM^ PerkinElmer Life Sciences, Monza, Italy) was measured to evaluate CDC. Viability was assessed on the same wells by addition of 10 µl of Resazurin (Celltiterblue reagent, Promega) in 100 µl of complete medium; fluorescence (485em/527ex, Victor3^TM^) was recorded after a 2 h incubation period.

Serum cytotoxicity was also measured by Flow Cytometry Complement-Cytotoxicity Assay^24^. Briefly, 50 µl of 1:4 diluted HI- sera were incubated for 30 min at 37 °C with detached CTPs. Thereafter, the cells were washed twice and incubated for 30 min at 37 °C with 1:10 diluted baby rabbit complement (Cederlane). Then, cells were washed and propidium iodide (1 µg ml^−1^) was added to detect dead cells. Percentage of PI-positive cells was recorded by FACScalibur cytometer (BD Bioscience) acquisition and analyzed by CELLQUEST software (BD bioscience). The data were evaluated by plotting FSC versus SSC, and the histograms with PI positive cells were analyzed. Experiments where PI-positive cells in negative control samples (i.e., untreated cells or cells treated with complement only) exceeded 12% of the R1-gated population were repeated. This gating strategy is illustrated in Supplementary Fig. 10b.

### Analyses of soluble mediators

A series of cytokines (IL-6, IL8, INF-y, TNF-α), chemokines (CCL2/MCP-1, CCL3/MIP-α, CCL4/MIP-β, CCL5/RANTES, CCL11/Eotaxin, CXCL10/IP-10, CXCL11/I-TAC, MIG, CXCL13/BLC, CXCL12/SDF-1α) and the neurotrophin BDNF have been evaluated in the sera and CSF of the CTP recipients at baseline and at different time-points following transplantation. These analytes were evaluated using a customized multiplex assay (Procartaplex, Multiplex Immunoassay, Affymetrix, Milan, Italy) by Luminex platform (Labscan100, Onelambda, Milan, Italy) in accordance with the manufacturer’s instructions. YKL-40 levels were determined for all the CSF samples using the Microvue CHI3L1 ELISA kit (Quidel Corporation, San Diego, CA) according to the manufacturer’s protocol. Absorbance was measured using a microplate reader (Bio-Rad).

### Reporting summary

Further information on research design is available in the Nature Research Reporting Summary linked to this article.

## Supplementary information

Supplementary Information

Peer Review File

Reporting Summary

## Data Availability

The data that support the findings of this study are available from the corresponding author upon reasonable request.
