# Supplementary material for: MHC matching fails to prevent long-term rejection of iPSC-derived neurons in non-human primates
Source: Nat Commun. 2019 Sep 25;10:4357. doi: 10.1038/s41467-019-12324-0 (PMC6761126; doi:10.1038/s41467-019-12324-0)
Supplement: Supplementary file 1 — Supplementary Information [file 41467_2019_12324_MOESM1_ESM.pdf]

MHC matching fails to prevent long-term rejection of iPSC-derived neurons in non-human primates

Romina Aron Badin et al.

Supplementary information

Cell therapy product	Monkey ID	Survival in months	MHCI & II genotype		Type of recipient
CTP_Mac1	Mac 1	3	M1	M1	Autologous (AU)
CTP_Mac2	Mac 2	6	M3	M3	Autologous (AU)
CTP_Mac3	Mac 3	3	M2	M5	Autologous (AU)
CTP_Mac1	Mac 4	6	M1	**Rec M3-M7**	Matched (MA)
CTP_Mac2	Mac 5	6	M3	**Rec M3-M1**	Matched (MA)
CTP_Mac1	Mac 6	3	M2	Rec M4-M1	Mismatched (MI)
CTP_Mac3	Mac 7	3	M6	M4	Mismatched (MI)

Supplementary Table 1: Summary of MHC I & II genotyping results of all grafted NHPs in the study, the cell therapy products (CTPs) received, type of graft recipient and the survival time post-grafting (PG).

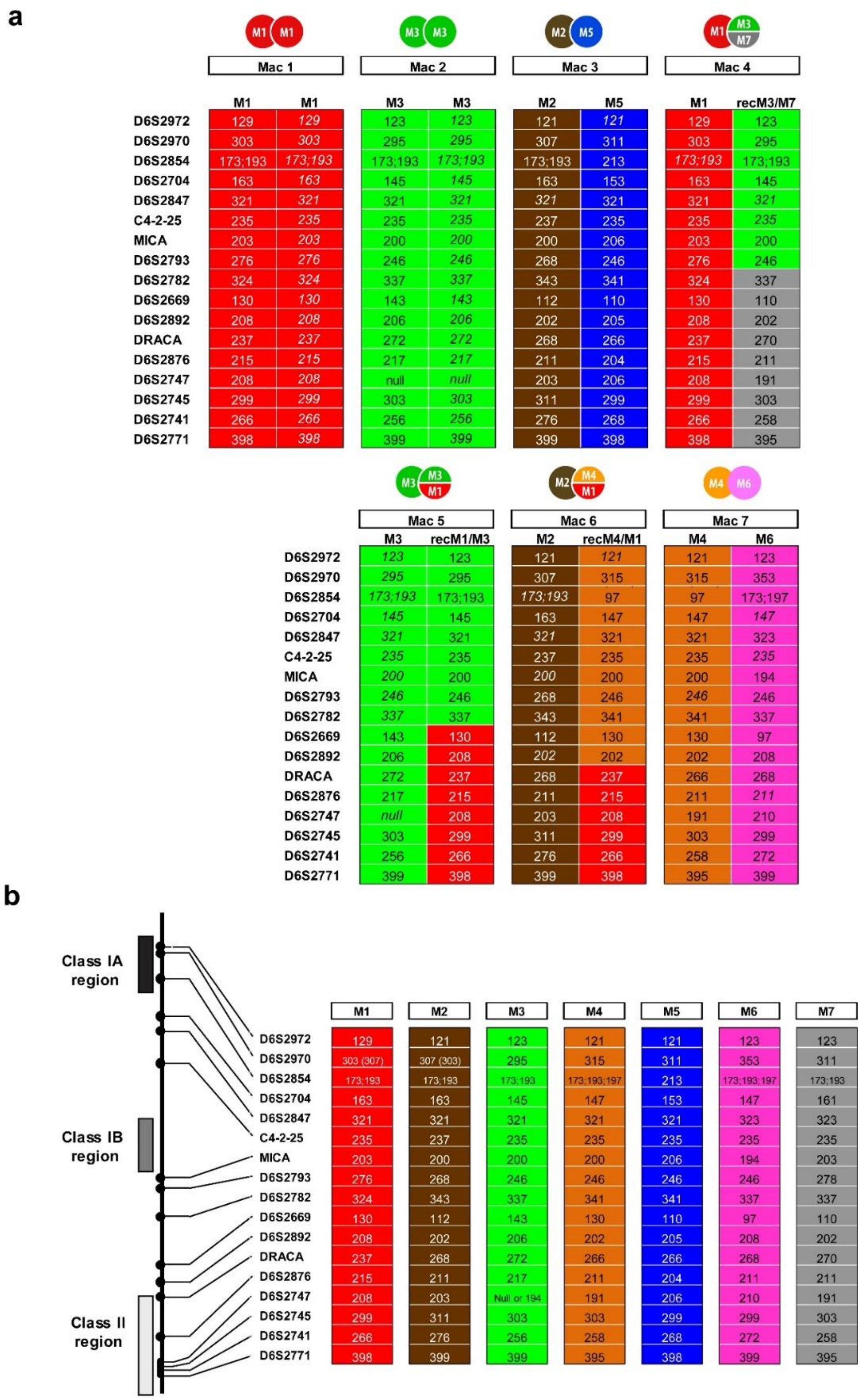

Supplementary Figure 1: Microsatellite Mafa haplotyping in cynomolgus NHPs. (a) Genomic DNA was extracted from blood samples collected from all macaques in the study. MHC genotyping was performed by studying 17 microsatellites spanning the entire MHC region. (b) The most probable correspondence with combinations of the seven ancestral Mauritian MHC haplotypes (M1-M7) (shown in b) was determined.

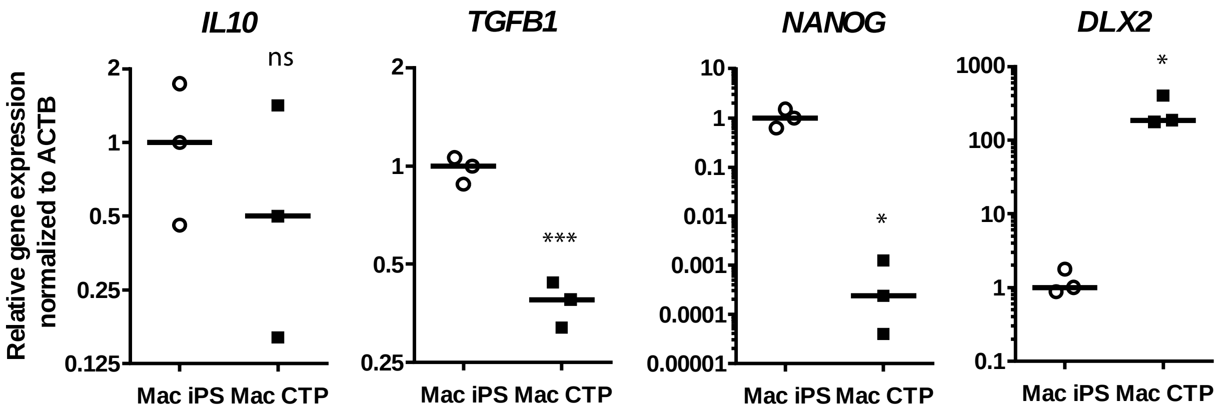

Supplementary Figure 2: The expression of *IL10* and *TGB1* in NHP CTPs. Quantification by qRT-PCR of *IL10*, *TGFB1*, *NANOG* (iPS marker), and *DLX2* (striatal marker) gene expression in macaque undifferentiated iPS (Mac_iPS) cells and in differentiated CTPs (Mac_CTPs). (n=3 biologically independent samples per condition, unpaired T- test, mean ± sem; *P<0.05; *** P<0.001).

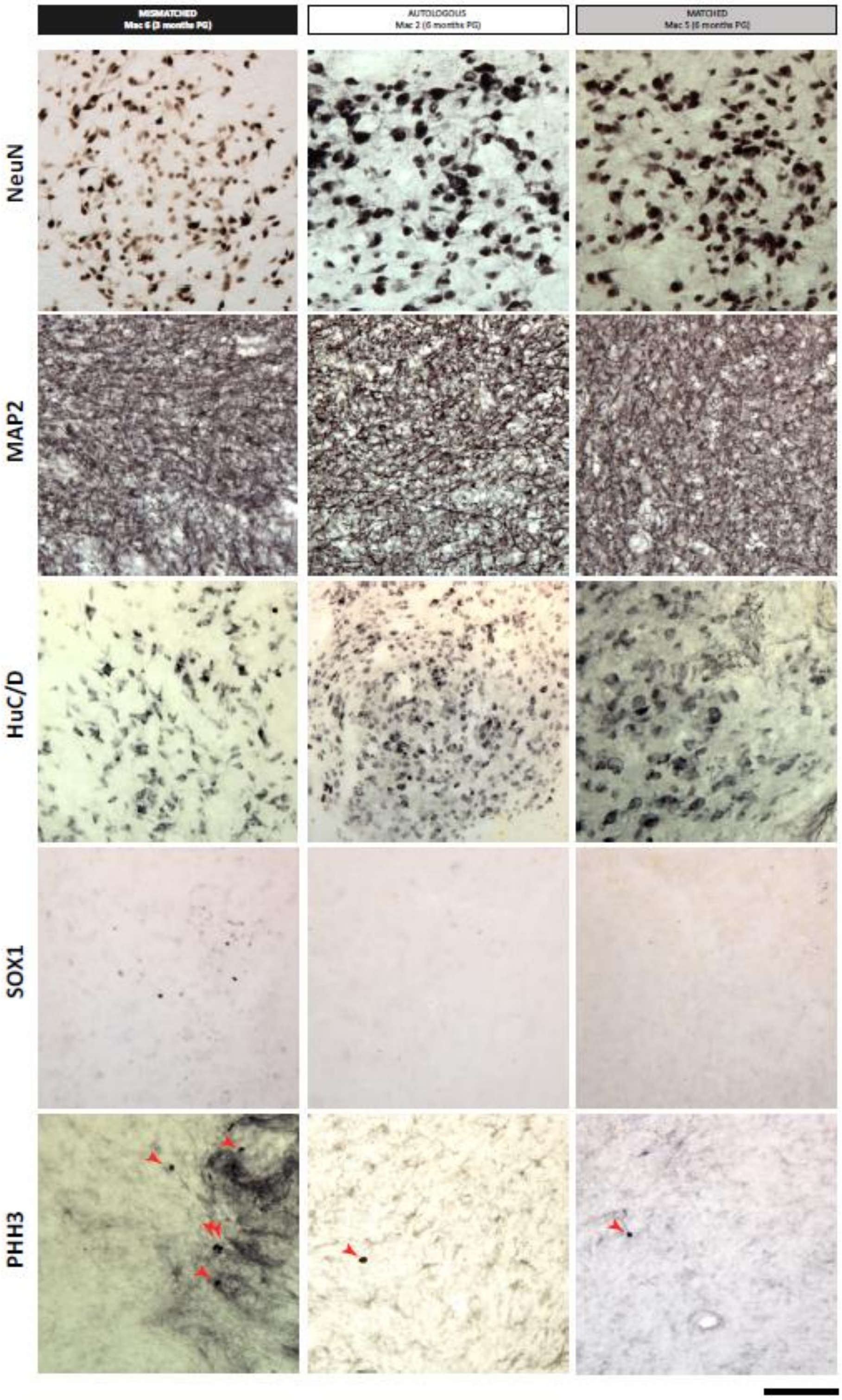

Supplementary Figure 3: Representative immunostainings showing the cellular composition of the grafts in mismatched (3 months PG), autologous (6 months PG) and matched (6months PG) NHP recipients. Brain slices were stained with post-mitotic neuronal markers NeuN (cell nuclei), MAP2 (soma and neuritic extensions), HuC/D (peri-nuclear soma), SOX1 (immature neural cells), and PHH3 (proliferative cells). Red arrowheads indicate PHH3-positive mitotic nuclei. Scale bar: 100 μm.

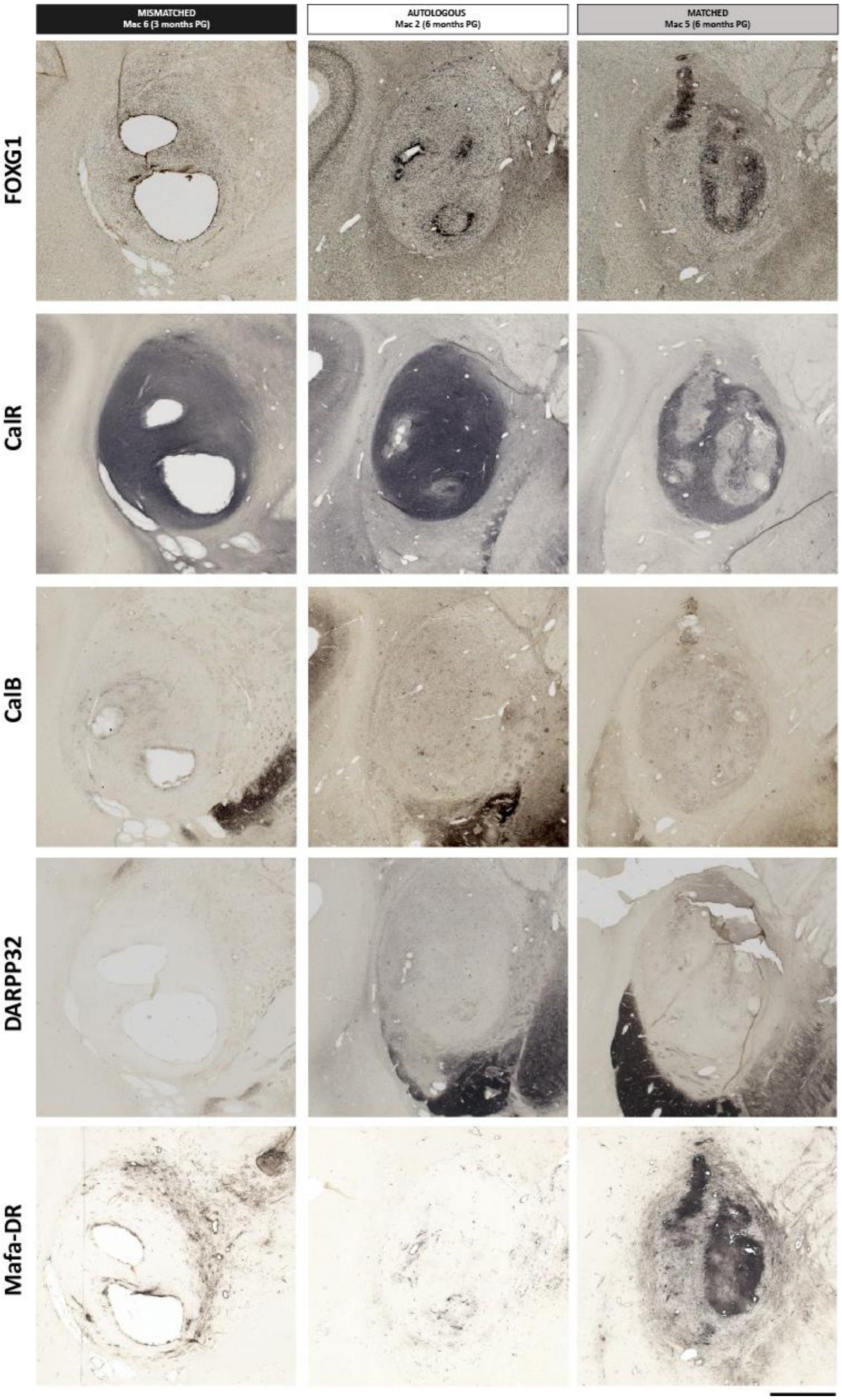

Supplementary Figure 4: Representative immunostainings showing the striatal identity profile of the grafts in mismatched (3 months PG), autologous (6 months PG) and matched (6 months PG) NHP recipients. Brain slices were stained with striatal markers including FOXG1 (telencephalic), calretinin (CalRet: interneurons), and Calbindin and DARPP32 (CalB, DARPP32: projection neurons) compared to Mafa-DR (MHC II). Scale bar: 1 mm.

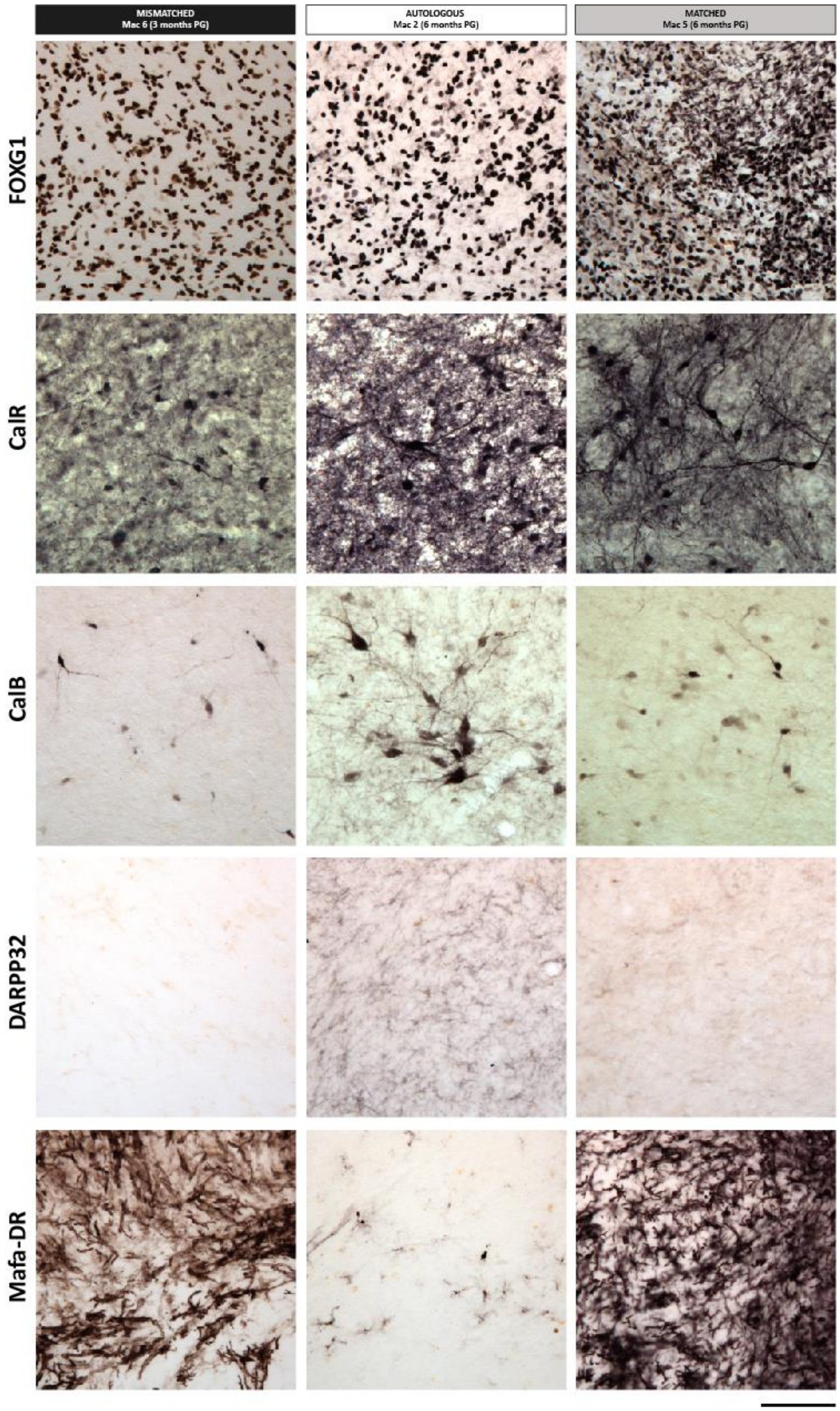

Supplementary Figure 5: Representative immunostainings showing the striatal identity profile of the grafts in mismatched (3 months PG), autologous (6 months PG) and matched (6 months PG) NHP recipients. Brain slices were stained with striatal markers including FOXG1 (telencephalic), calretinin (CalRet: interneurons), and Calbindin and DARPP32 (CalB, DARPP32: projection neurons), compared to Mafa-DR (MHC II). Scale bar: 100 µm.

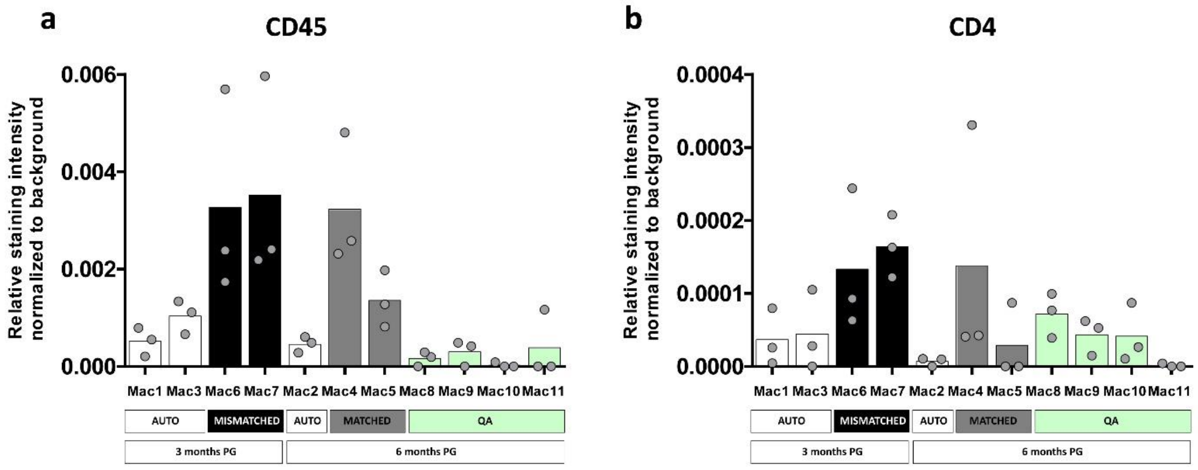

Supplementary Figure 6: Post-mortem analysis of CD45 and CD4-positive cells. Quantification of CD45 (a) and CD4 (b) immunoreactivity in AU, MA, MI CTP recipients at 3 or 6 months post-grafting (PG), and in untransplanted controls to show the contribution of the QA-induced lesion to the staining in lesioned and grafted NHPs. Bar graphs represent mean value of the three regions of interest considered (left and right caudate and left putamen) for each animal; grey dots represent individual values for each region (n=3 regions per animal).

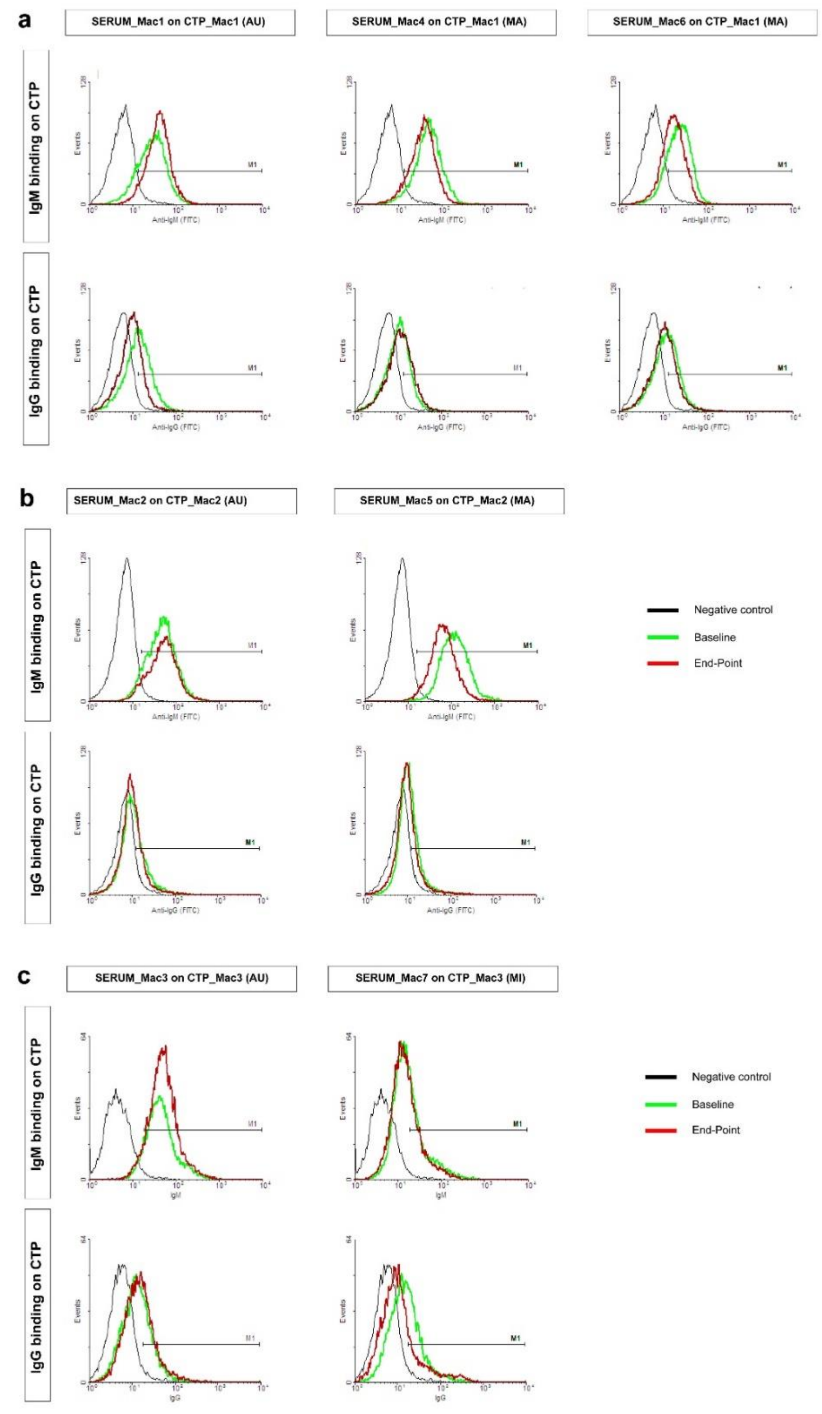

Supplementary Figure 7: Detection of anti-CTP-specific antibodies by flow cytometry. The binding levels of IgM and IgG to CTPs were assessed at baseline (green histograms) and at the end-point (euthanasia, red histograms) using Flow Cytometry. Each CTP was incubated with recipient serum followed by FITC-conjugated anti-IgM or IgG. Black histogram- secondary antibody only (negative control).

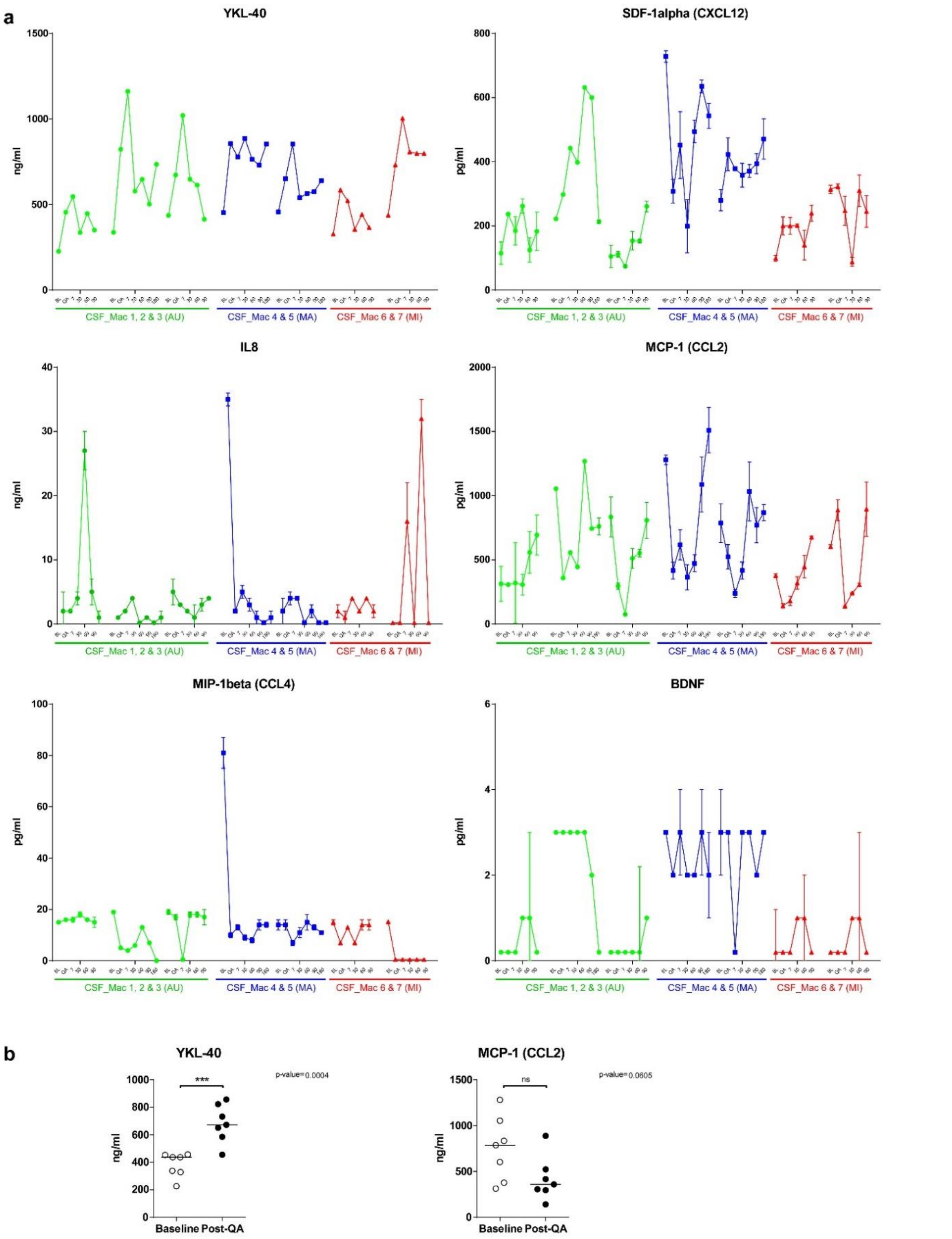

Supplementary Figure 8: Longitudinal measurement of 6 cytokines (YKL-40, IL8, MCP-1, MIP1β, SDF1a and BDNF) in the CSF of AU (green), MA (blue) and MI (red) recipients. (a) Measurements at baseline (BL), post-QA (QA) and 7, 30, 60, 90, 180 days post-grafting. (n=2 per time point, mean ± sd) (b) Comparison of YKL-40 and MCP-1 at baseline and post-QA lesion (n=7 biologically independent samples per group, unpaired T- test, *** P<0.001).

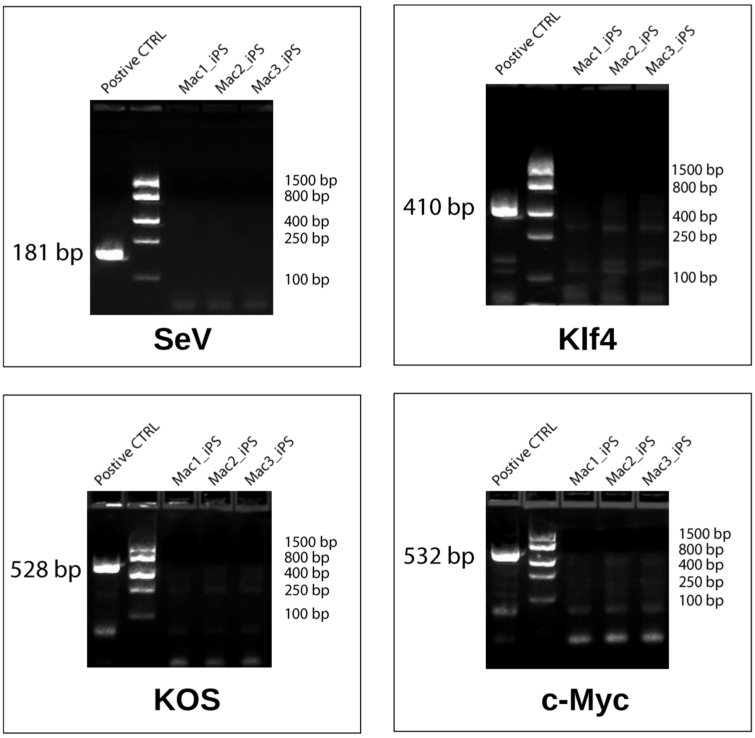

Supplementary Figure 9: In vitro quality control of NHP iPSCs to assess the absence of SeV genome and transgenes.

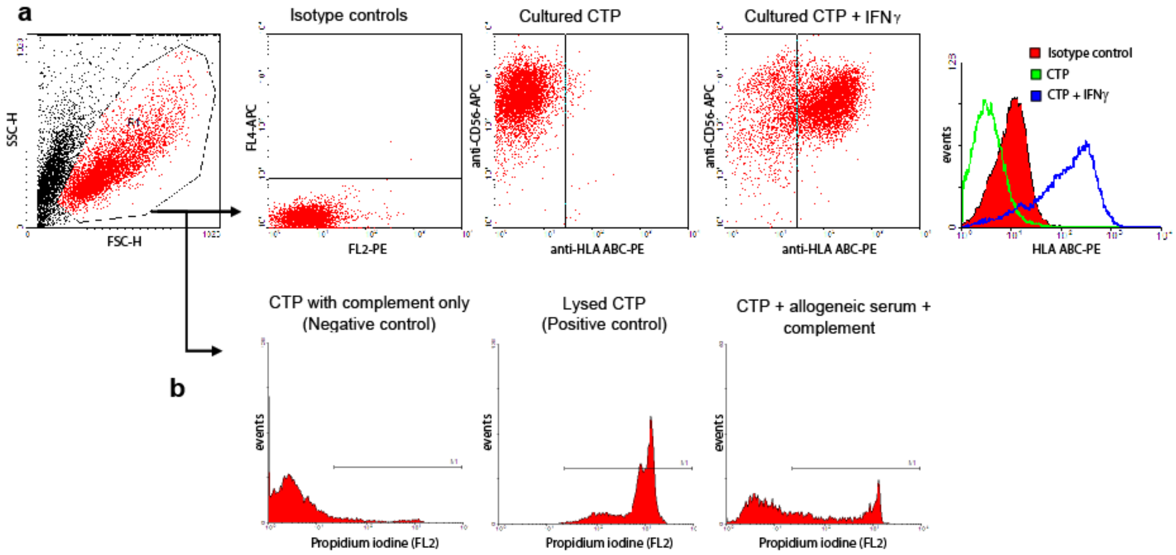

Supplementary Figure 10: Gating strategy in immunophenotyping experiments. (a) The figure shows a representative example of data gating following immunostaining with the anti-HLA class I antibody clone I3,9-49 (BeckmanCoulter Life Sciences, Milan, Italy). A R1 region was defined on forward scatter (FSC)/ side scatter (SSC) biparametric histograms. Negative controls were defined following labelling with isotype controls. Cells included in R1 region were found positive for the CD56 NCAM marker. Control histograms (isotype controls) were overlaid onto the stained positive dataset allowing positive cells to be accurately identified on single parameter histograms. (b) Sample gating strategy for determining complement-mediated cell lysis. Histograms showing the PI staining of CTPs treated with complement only (negative control), with lysis solution (positive control) or with the allogeneic serum and complement are reported as an example.
