# Supplementary material for: MHC matching fails to prevent long-term rejection of iPSC-derived neurons in non-human primates
Source: Nat Commun. 2019 Sep 25;10:4357. doi: 10.1038/s41467-019-12324-0 (PMC6761126; doi:10.1038/s41467-019-12324-0)
Supplement: Supplementary file 3 — Reporting Summary [file 41467_2019_12324_MOESM3_ESM.pdf]

nature research

Corresponding author(s): Anselme Perrier

Last updated by author(s): 7/25/2019

Reporting Summary

Nature Research wishes to improve the reproducibility of the work that we publish. This form provides structure for consistency and transparency in reporting. For further information on Nature Research policies, see Authors & Referees and the Editorial Policy Checklist.

Statistics

For all statistical analyses, confirm that the following items are present in the figure legend, table legend, main text, or Methods section.

n/a	Confirmed	
☐	☒	The exact sample size (*n*) for each experimental group/condition, given as a discrete number and unit of measurement
☐	☒	A statement on whether measurements were taken from distinct samples or whether the same sample was measured repeatedly
☐	☒	The statistical test(s) used AND whether they are one- or two-sided<br>*Only common tests should be described solely by name; describe more complex techniques in the Methods section.*
☒	☐	A description of all covariates tested
☐	☒	A description of any assumptions or corrections, such as tests of normality and adjustment for multiple comparisons
☐	☒	A full description of the statistical parameters including central tendency (e.g. means) or other basic estimates (e.g. regression coefficient) AND variation (e.g. standard deviation) or associated estimates of uncertainty (e.g. confidence intervals)
☒	☐	For null hypothesis testing, the test statistic (e.g. *F*, *t*, *r*) with confidence intervals, effect sizes, degrees of freedom and *P* value noted<br>*Give P values as exact values whenever suitable.*
☒	☐	For Bayesian analysis, information on the choice of priors and Markov chain Monte Carlo settings
☒	☐	For hierarchical and complex designs, identification of the appropriate level for tests and full reporting of outcomes
☒	☐	Estimates of effect sizes (e.g. Cohen's *d*, Pearson's *r*), indicating how they were calculated

Our web collection on statistics for biologists contains articles on many of the points above.

Software and code

Policy information about availability of computer code

Data collection	Mercator (Exploranova, La rochelle, France) for quantification of immunohistochemical data
Data analysis	GraphPad Prism for statistical analysis and graphs

For manuscripts utilizing custom algorithms or software that are central to the research but not yet described in published literature, software must be made available to editors/reviewers. We strongly encourage code deposition in a community repository (e.g. GitHub). See the Nature Research guidelines for submitting code & software for further information.

Data

Policy information about availability of data

All manuscripts must include a data availability statement. This statement should provide the following information, where applicable:

- Accession codes, unique identifiers, or web links for publicly available datasets
- A list of figures that have associated raw data
- A description of any restrictions on data availability

Provide your data availability statement here.

Field-specific reporting

Please select the one below that is the best fit for your research. If you are not sure, read the appropriate sections before making your selection.

☒ Life sciences ☐ Behavioural & social sciences ☐ Ecological, evolutionary & environmental sciences

For a reference copy of the document with all sections, see nature.com/documents/nr-reporting-summary-flat.pdf

Life sciences study design

All studies must disclose on these points even when the disclosure is negative.

Sample size	In the case of non-human primate studies it is rare to use standard power calculations for ethical and cost reasons. The sample size presented in this paper is in agreement to the reported sample sizes in the litterature in other studies using non-human primates.
Data exclusions	No data exclusions
Replication	The work presented in this study was acquired in non-human primates and in view of 3R principles and ethical reasons, no replication experiments were performed. Measurement replications (e.g. on peripheral fluids) have been recplicated and indicated inthe manuscript as appropriate
Randomization	No radomization applied since animals had to be gentoyped to be allocated in to the different treatment groups
Blinding	No blinding performed

Reporting for specific materials, systems and methods

We require information from authors about some types of materials, experimental systems and methods used in many studies. Here, indicate whether each material, system or method listed is relevant to your study. If you are not sure if a list item applies to your research, read the appropriate section before selecting a response.

Materials & experimental systems

n/a	Involved in the study	
☐	☒	Antibodies
☐	☒	Eukaryotic cell lines
☒	☐	Palaeontology
☐	☒	Animals and other organisms
☒	☐	Human research participants
☒	☐	Clinical data

Methods

n/a	Involved in the study	
☒	☐	ChIP-seq
☐	☒	Flow cytometry
☐	☒	MRI-based neuroimaging

Antibodies

Antibodies used	DARPP-32 1:100 (2302 Cell signaling, Leiden, Netherlands); NeuN 1:5000 (MAB377, Millipore, Billerica, MA); FOXG1 1:500 (AB18259, Abcam, Cambridge, UK), iBA1 1:1000 (1919741,Wako, Osaka, Japan), CD8 1:100 (A07757, Beckman Coulter, Villepinte, France.), MHC-Class II 1:500 (MO746, DakoCytomation, Glostrup, Denmark), CD68 1:5000 (M0718, DakoCytomation, Glostrup, Denmark), CD4 1:800 (M7310, DakoCytomation, Glostrup, Denmark), CD45 1:1000 (M0701, DakoCytomation, Glostrup, Denmark), Calretinin 1:5000 (AB5054, millipore, Billerica, MA), Calbindin1:1000 (C9848, Sigma, st Louis, MO, US), PHH3 1:1000 (06-570, Millipore, Billerica, MA), GFAP 1:5000 (Z0334, DakoCytomation, Glostrup, Denmark), MAP2 1:800 (M1406, Sigma), FoxP1 1:800 (Ab32010, AbCam, Cambridge, UK), HNA 1:400 (mab1281, Millipore, Billerica, MA), OCT3/4 1:200 (sc5279, Santa Cruz, Dallas, Texas), Sox1 1:200 (4194, Cell signalling, Danver, MA), HuC/HuD 1:500 (A-2127, ThermoFisher, Waltham, MA)
Validation	DARPP-32 Antibody detects endogenous levels of total human DARPP-32 independent of phosphorylation.<br>NeuN antibody (NEUronal Nuclei; clone A60) specifically recognizes the DNA-binding, neuron-specific protein NeuN, which is present in most CNS and PNS neuronal cell types of all vertebrates tested. Specificity validated in human by the manufacturer.<br>FOXG1 Expression is restricted to the neurons of the developing telencephalon. Antibody directed against a synthetic peptide corresponding to Human FOXG1 aa 400 to the C-terminus<br>iBA1 These products are antibodies that specifically recognize Iba1, and are available for a microglial marker. Specificity validated in human by the manufacturer.<br>CD8: The B9.11 monoclonal antibody recognizes the α chain of the human CD8 molecule<br>MHC Class II Immunogen: 33 kDa a-chain subunit of HLA-D products prepared from the B lymphoblastoid cell line Bristol 8, The antibody reacts with B cells, activated T cells, macrophages, antigen presenting cells<br>CD68. Immunogen: Macrophages isolated from human lung<br>CD4. Immunogen: Recombinant protein corresponding to the external domain of the human CD4 molecule. This antibody labels thymocytes and T-helper cells (1)<br>Anti-CD45 is a mixture of two monoclonal antibodies, clones 2B11 and PD7/26 directed against human proteins. Clone 2B11 reacts with all the known isotypes of the CD45 family and clone PD7/26 was clustered as anti-CD45RB<br>Calretinin. Specific for calretinin, validated in rat brain cortex and human cerebellum by the manufacturer.<br>Calbindin. The antibody is specific for the calbindin 28K and does not react with other members of the EF-hand family. Specificity validated in human by the manufacturer<br>PHH3. Anti-phospho-Histone H3 (Ser10) Antibody is a mitosis marker by detection of human Histone H3 phosphorylated at serine 10<br>GFAP Glial fibrillary acidic protein is expressed in astrocytes.<br>The MAP2 antibody is the major microtubule associated protein of brain tissue and labelled the post-mitotic neurons.<br>FoxP1 labels medium spiny neurons (MSN) in vitro and is required for DARPP-32+ MSN differentation.

HNA: The antibody is human-specific nuclei.
OCT3/4 is a transcription factor frequently used as a marker for undifferentiated cells.
Sox1 is one of the earliest markers that identify neuroectodermal tissue and mark neuronal stem cells
HuC/HuD is a marker of neuronal cells in tissue

Eukaryotic cell lines

Policy information about cell lines

Cell line source(s)	IPSC derived from Macaca fascicularis PBMC
Authentication	None of the cell lines used were authenticated
Mycoplasma contamination	We confirm that all cell lines tested negative for mycoplasma
Commonly misidentified lines (See ICLAC register)	No commonly misdentified cell lines used in this study

Animals and other organisms

Policy information about studies involving animals; ARRIVE guidelines recommended for reporting animal research

Laboratory animals	Rodent studies<br>Species: Rat<br>strain: NIH nude (Charles Rivers)<br>sex : female<br>age: 10-12 weeks (young adults)<br>NHP studies<br>Species: Macaca fascicularis<br>Strain: N/A<br>Sex: male<br>Age: mean 5,4 +:- 0,2 (adult)
Wild animals	This study did not involve wild animals
Field-collected samples	This study did not involve field collected samples
Ethics oversight	Ethical approval of the experimental protocol was obtained from the local ethics committee CETEA n°44 and the Ministry of Research and Higher Edication (authorization n°14_019).

Note that full information on the approval of the study protocol must also be provided in the manuscript.

Flow Cytometry

Plots

Confirm that:

☒ The axis labels state the marker and fluorochrome used (e.g. CD4-FITC).

☒ The axis scales are clearly visible. Include numbers along axes only for bottom left plot of group (a 'group' is an analysis of identical markers).

☐ All plots are contour plots with outliers or pseudocolor plots.

☐ A numerical value for number of cells or percentage (with statistics) is provided.

Methodology

Sample preparation	Immunophenotyping of cell therapy products (CTPs): The primate CTP lines examined in these studies were cultured on poly-ornithin laminin-coated 48-well plates at 105 cells/cm2 . Culture medium with or without INF-γ (100 ng/ml) was added for 48hrs prior to cell harvesting with Accutase (StemPro Accutase, Thermo Scientific, Waltham, MA). Briefly, 105 cells were incubated with PE-labelled antibodies [HLA A, B, C (clone G46-2.6, BD-Pharmingen, San Diego, CA); HLA-DP, DQ, DR (clone I3,9-49, BeckmanCoulter Life Sciences, Milan, Italy); CD40 (clone 5C3), CD80 (clone L307.4), CD86 (clone FUN-1) (all from BD-Pharmingen)] for 30 minutes at 4°C, washed with phosphate-buffered saline (PBS), 0.5% bovine serum albumin (BSA), and 0.1% Na-Azide.<br>Anti-graft antibody response: The anti-graft antibody response was confirmed by flow cytometry. Briefly, cultured donor CTPs were detached with Accutase (StemPro Accutase, Thermo Scientific, Waltham, MA) and incubated for 30 minutes at 37°C with heat-inactivated (HI) recipient sera (diluted with PBS/BSA/Na-azide). After washing with PBS-BSA-sodium azide, antibody binding was revealed using FITC-labelled anti-human IgG (Jackson ImmunoResearch Laboratories, Sacramento, CA) or IgM (Dako, Milan, Italy) antibodies. Incubation with the secondary only was used as a negative control.<br>Complement-Mediated Cytotoxicity Assay: Briefly, 50µl of 1:4 diluted HI- sera were incubated for 30 min. at 37°C with detached CTPs (105 cells). CTPs were washed twice and incubated for 30 min at 37°C with 1:10 diluted rabbit complement (Cederlane, Burlington, ON). Subsequently, cells were washed twice and propidium iodide (1µg/ml) was added to detect dead cells.

Instrument	Flow cytometric data acquisition was performed on a FACScalibur flow cytometer (Becton Dickinson, San Jose, CA)
Software	Median fluorescence intensity (MFI) and Percentage of PI-positive cells were analyzed by CELLQUEST software (BD Bioscience).
Cell population abundance	No sorting experiments were performed. Data were generated using detached cultured donor CTPs, collecting 20,000 events on R1 region. The R1 region includes at least 90% of total events collected.
Gating strategy	The data have been analysed by plotting forward scatter (FSC) versus side scatter (SSC) and by defining a R1 region that includes at least 90% of events collected.<br>Experiments of CTPs immunophenotyping and anti-graft antibody detection: control histograms (isotype controls) were overlaid onto the stained positive dataset allowing positive cells to be accurately identified on single parameter histograms (PE-FL2 or FITC-FL1 fluorescence). The lower limit of markers for the positive population were set to include less than 2% of events in the isotypic control (negative control).<br>Experiments of Complement-Mediated Cytotoxicity: the gating strategy applied was that previously reported by Duensing, T. D. & Watson, S. R. (Complement-Dependent Cytotoxicity Assay. Cold Spring Harbor protocols 2018, pdb prot093799, doi:10.1101/pdb.prot093799 (2018)". The data were analysed by plotting forward scatter (FSC) versus side scatter (SSC), and the histograms with PI positive cells were analysed. Experiments where PI-positive cells in negative control samples (i.e. untreated cells or cells treated with complement only) exceeded 12% of the R1- gated population were repeated.

☐ Tick this box to confirm that a figure exemplifying the gating strategy is provided in the Supplementary Information.

Magnetic resonance imaging

Experimental design

Design type	Anatomical
Design specifications	T2-weighted images taken at monthly intervals until end-point
Behavioral performance measures	N/A

Acquisition

Imaging type(s)	Anatomical
Field strength	7T
Sequence & imaging parameters	T2-weighted images were acquired using a high-resolution 2D fast spin-echo sequence (469×469 μm² in-plane resolution, 1 mm slice thickness, 70 slices), with echo time TE/ Repetition time TR = 20/8000 ms, 5 echoes, effective TE = 52.5 ms and acquisition time Tacq = 43 min. For T2*-weighted images the parameters used were: 469×469 μm² in-plane resolution, 1 mm slice thickness, 40 slices, 5 TE (from 5.5 to 30 ms), repetition time TR = 2 ms and acquisition time Tacq = 8 min.
Area of acquisition	whole brain

Diffusion MRI ☐ Used ☒ Not used

Preprocessing

Preprocessing software	N/A
Normalization	N/A
Normalization template	N/A
Noise and artifact removal	N/A
Volume censoring	N/A

Statistical modeling & inference

Model type and settings	N/A
Effect(s) tested	N/A

Specify type of analysis: ☐ Whole brain ☐ ROI-based ☐ Both

Statistic type for inference (See Eklund et al. 2016)	N/A
Correction	N/A

Models & analysis

n/a	Involved in the study
☒	☐ Functional and/or effective connectivity
☒	☐ Graph analysis
☒	☐ Multivariate modeling or predictive analysis
