# Supplementary material for: MHC matching fails to prevent long-term rejection of iPSC-derived neurons in non-human primates
Source: Nat Commun. 2019 Sep 25;10:4357. doi: 10.1038/s41467-019-12324-0 (PMC6761126; doi:10.1038/s41467-019-12324-0)
Supplement: Supplementary file 2 — Peer Review File [file 41467_2019_12324_MOESM2_ESM.pdf]

Reviewers' comments:

Reviewer #1 (Remarks to the Author):

In contrast to previous reports indicating that cells derived from non-human primate iPSCs are not immune rejected in HLA-matched normal recipients, the authors concluded that neural cells derived from non-human primate iPSCs are immune rejected when transplanted into the lesion sites of HLA-matched disease models for Huntington's disease. Because the disease model better recapitulates the scenario of human cell therapy, this conclusion is more clinically relevant. However, there are a number of concerns that need to be addressed before this manuscript can be considered for publication.

1. The authors should clarify the number of animals analyzed for each dataset in the figure legends including the supplementary figures. It is not clear to this reviewer whether the number of animals used is sufficient to draw the conclusion.

2. The authors should provide some data on how various transplants rescue the disease phenotypes, a key readout of the successful cell transplantation.

3. While the data of CD8 staining show that HLA-matched grafts are extensively infiltrated with CD8+ T cells, there are also lower levels of CD8+ T cell infiltration in the autologous grants, making it difficult to draw the conclusion that autologous grafts are not immunogenic. The authors should provide data on the infiltrating CD4+ T cells and macrophages that can mount delayed type immune rejection of grafts. The levels of various infiltrating immune cells should be quantified and statistically analyzed.

4. There is no data to compare the immune responses to HLA-mismatched and matched grafts. This data is important to understand any benefits of HLA-matching in iPSC-based cell therapy.

5. The authors should present data on whether the iPSCs are integration free (no random integration of reprogramming vector into the genome). The abnormal expression of reprogramming factors is known to induce immunogenicity of iPSC-derived cells.

6. Based on previous findings that endothelial cells derived from mouse iPSCs can be immune tolerated by syngeneic recipients due to overexpression of immune suppressive cytokine IL-10, the authors should analyze the expression of immune suppressive cytokines in neural cells derived from iPSCs.

7. In the introduction, some information on previous studies of the immunogenicity of iPSC-derived cells is incorrect. For example, the humanized mouse studies have shown that certain human iPSC derived cells are robustly immune rejected.

Reviewer #2 (Remarks to the Author):

The study by Badin et al addresses a issue of great current interest in the cell therapy field. Understanding of how host immune responses to iPSC-derived cell products, transplanted to the brain, can be controlled by immune matching will be of great value in the planning of future clinical trials. From that point of view this paper is a valuable and welcome contribution. However, the study has several shortcomings that need to be dealt with before it can be considered for publication.

1. The presentation of the histological data from the transplanted animals needs to be

complemented with high power pictures showing the cellular composition of the transplants. From the low magnification pictures shown in Fig 1C it seems that the grafts are quite heterogenous in composition with large numbers of immature-looking cells, suggesting that they have expanded considerably in size over the 6 months period. For this reason it would be essential to include an analysis of the extent of cellular proliferation at the three time points, 1, 3 and 6 months using eg PCNA staining. This is important since continuously expanding grafts are likely to contain areas where the BBB remains immature and leaky. Similarly, the MR images in Fig 1B are too small to allow the reader to evaluate the images. The relevant hyper-intense areas should be indicated.
It is notable that grafts are placed in both caudate and putamen (as mentioned in Methods), but this is not properly commented on in the results section. They should be independently labeled in the figures.

2. It is important that the procedure used to generate the MHC matched cells is described in detail. I am not an immunologist, but I find it unsatisfactory that the exact description of which MHC genes/Mafa haplotypes have been selected for matching is lacking, and whether the resultant cell line was homozygous for these genes.

3. The discussion fails to deal with several issues of importance for the interpretation of the reported (negative) results. In particular, the differences between the extent of MHC matching in this study and the one used in the recent Morizane et al paper need to be discussed in detail. Were the same Mafa genes targeted for matching in both studies, and to what extent did the Mafa haplotypes match the ones expressed by the monkeys used in the transplantation experiment in this study? (In the Morizane study the receipient monkeys had at least one of the alleles identical to the MHC matched transplants). To make the discussion really interesting, the authors should include a discussion of the shortcoming(s) of the degree of MHC matching used in this study, and how it could be improved in order to obtain graft cells that are sufficiently well matched to evade the immune response. A detailed comparison with the procedure and matching used in the Japanese study may shed light on this interesting question.

4. Minor comment: In the discussion of the reasons for the different results obtained in this and the Morizane et al study I suggest that the authors should add one more factor that may be of particular importance, namely that grafts that expand in size after transplantation, due to continued cell division of more immature cells, may retain areas with immature or leaky BBB and thus be more prone to trigger the host´s immune response.

Reviewer #3 (Remarks to the Author):

This is a difficult paper to evaluate. Firstly these are difficult to perform experiments that often will never be repeated and thus the data provide value to the scientific community. However the study is difficult to interpret within the context of grafting of striatal IPSC's for Huntingtons's disease. Prior studies, as acknowledged by the authors demonstrate successful grafting with unlesioned animals. The present study performed similar grafting in quinolinic acod lesioned mokneys. The question is, how representative is that in terms of the necrotic environment the QA lesions causes to that of HD victims. The answer is the QA lesion provides a far more violent environment than what is seen in HD with liquifactive necrosis, wholes in the tissue and robust inflammation. Therefore is this model a fair test for the question under study. I wonder if the same result would be found with a more gentle lesion such as the other lesoining methods this outstanding group has published in the past.

POINT-BY-POINT RESPONSE

Reviewers' comments:

Reviewer #1 (Remarks to the Author):

In contrast to previous reports indicating that cells derived from non-human primate iPSCs are not immune rejected in HLA-matched normal recipients, the authors concluded that neural cells derived from non-human primate iPSCs are immune rejected when transplanted into the lesion sites of HLA-matched disease models for Huntington's disease. Because the disease model better recapitulates the scenario of human cell therapy, this conclusion is more clinically relevant. However, there are a number of concerns that need to be addressed before this manuscript can be considered for publication.

1. The authors should clarify the number of animals analyzed for each dataset in the figure legends including the supplementary figures. It is not clear to this reviewer whether the number of animals used is sufficient to draw the conclusion.

Following the reviewer's comment, we have now added n numbers more clearly in the text of the manuscript and in every figure legend (including supplementary material) to clarify this point. We compared 4 QA controls euthanized at 6 months post-lesioning to 7 transplanted animals euthanized at 3 (n=4) and 6 months (n=3) post-grafting. We agree with the reviewer that the n numbers are low. However, in accordance with the 3R principle and in line with the ethical requirements underlying animal research with NHP, we reduced the number of animals used to the minimum. Moreover, after a quick survey of the literature dealing with cell transplantation in the brain or eye of NHPs, we confirm that our numbers are comparable to those in recently published papers, accepted in highly ranked journals. In particular, please refer to:

Emborg et al, 2013 (two publication) (Emborg, Liu et al. 2013; Emborg, Zhang et al. 2013) (n=3); Morizane et al, Stem cell reports 2013 (Morizane, Doi et al. 2013) (n=4); Kikuchi et al (Takahashi lab), Nature 2017 (Kikuchi, Morizane et al. 2017) (n=3/4); Morizane et al, Nat Comm 2017 (Morizane, Kikuchi et al. 2017) (n=4/group); Kriks et al (Studer lab), Nature 2011 (Kriks, Shim et al. 2011) (n=2); Sugita et al, Stem cell reports 2017 (Sugita, Makabe et al. 2017) (n=2/3).

2. The authors should provide some data on how various transplants rescue the disease phenotypes, a key readout of the successful cell transplantation.

Although the primary aim of our work was to study the relationship between the immune response of the host and the transplanted cells, we understand the reviewer's concerns regarding a clinical readout. We believe that the prerequisite to evaluate a clinically pertinent cell therapy product is to demonstrate:

a) Survival & engraftment n: our grafted cells survive (in particular AU but also MA) up to 6 months post-transplantation in complete absence of peripheral immunosuppression

b) Graft size: the grafts obtained cover a good portion of the caudate and putamen and the heterogeneity observed is linked to the cell line used (clone) rather than inter-animal variability. This is an important parameter for scaling-up and QC criteria of cell therapy batches

c) Detection of adverse events: our MRI data enabled us to predict graft size (therefore also potential overgrowth) and vacuolation of grafts that were heavily attacked by the immune system, as evidenced by post-mortem immunohistochemistry. No overproliferation was observed in any of the cell therapy products used at 3 or 6 months post-grafting as confirmed by post-mortem analysis (Phospho-Histone H3 staining PHH3: see Refere#2, response 1)

d) Cell differentiation/maturation: at the early stages at which we euthanized the primates in this study we could observe that most cells were neuronal (see below)

e) Immunogenicity and inflammation

f) Functionality and connectivity of grafted cells

g) Functional biomarkers for follow-up.

The reviewer might agree that establishing the optimal parameters for each of these important issues in a single experiment is ambitious. We have, however, clearly shown that cells implant and survive; grafts have a good size in terms of striatal coverage and are not heterogeneous between animals grafted with the same cell line. From a safety point of view, cells do not overproliferate, they differentiate into striatal cells (interneurons), and are immunogenic in the absence of peripheral immunosuppression. From an immunological standpoint, maturation is key as immature cells could display antigenic residues on the surface that are absent on mature cells. In this light, we waited long enough after transplantation to ensure that the response to the transplant was directed to mature cells [i.e. post-mitotic striatal neurons (see PHH3+ cell counts)]. We performed longitudinal behavioral tests and end-point PET exams to explore the degree of cell differentiation; and used MRI as an indicator of ongoing rejection to decide the timepoint for euthanasia. Indeed, it was essential to ensure that we had appropriate tissue (i.e. grafts undergoing immune rejection as opposed to a glial scar in the absence of grafted cells) in order to perform this study. Unfortunately, and unlike in our rodent control transplants (Supplementary Fig. 3_R1, 6 months post-transplant were not sufficient to observe DARPP32 positive cells in our primates and, therefore, the assessment of the potential therapeutic benefit is not as pertinent. In future studies, in order to address this issue, we plan to both optimize the differentiation protocol to generate MSNs in primates and to wait longer after transplantation in peripherally immunosuppressed NHP.

3. While the data of CD8 staining show that HLA-matched grafts are extensively infiltrated with CD8+ T cells, there are also lower levels of CD8+ T cell infiltration in the autologous grafts, making it difficult to draw the conclusion that autologous grafts are not immunogenic. The authors should provide data on the infiltrating CD4+ T cells and macrophages that can mount delayed type immune rejection of grafts. The levels of various infiltrating immune cells should be quantified and statistically analyzed.

We thank the reviewer for this constructive comment.
We have taken the time to explore which populations of immune cells are present in our grafted primates. We have also added important information on <u>lesioned ungrafted control</u> animals that was not present in the first version of the manuscript.
Indeed, the reviewer appreciated that we were studying the effect of cell transplantation in an inflammatory environment (as it occurs in human HD) and not in a healthy brain.

We have now added quantification of several markers of immune cells in both "lesioned-and-grafted" and "lesioned-only" animals to tease out the contribution of the lesion per se to the inflammatory signal that is present as a "background reaction" in all grafted primates.
As it can be observed from the revised Fig. 2_R1 and the new supplementary Fig. 6_R1 we have quantified levels of CD8+ T cells, CD4+ T cells, CD45+ and CD68+ cells and in most cases the levels of expression detected in autologous grafted primates are similar to those detected in four QA lesioned-ungrafted controls. The mean signal in the same anatomical regions of interest of 4 QA-lesioned NHPs (non-transplanted control animals) has been quantified and is now indicated as a dashed line to illustrate QA-induced signals (in Fig2_R1 and Supplementary Fig.6_R1). These results suggest that the immune markers observed in the autologous grafts are due to the lesion itself rather than the grafted cells.

4. There is no data to compare the immune responses to HLA-mismatched and matched grafts. This data is important to understand any benefits of HLA-matching in iPSC-based cell therapy.

The experimental design used here was heavily influenced by unpublished data generated in the laboratory and published data (Emborg et al 2013, Cell transplantation (Emborg, Zhang et al. 2013) regarding mismatched grafts.

In fact, in a previous ANR-funded study we grafted 6 QA-lesioned macaques (identical lesion paradigm) with allogeneic iPSCs that (now we know) were totally mismatched. We allowed the animals to survive 6-7 months post-surgery in the absence of peripheral immunosuppression. We were able to retrieve only one graft out of six and this was heavily damaged and vacuolated, as seen on MRI images in vivo and post-mortem immunohistochemistry using and anti-GFAP antibody (see below).

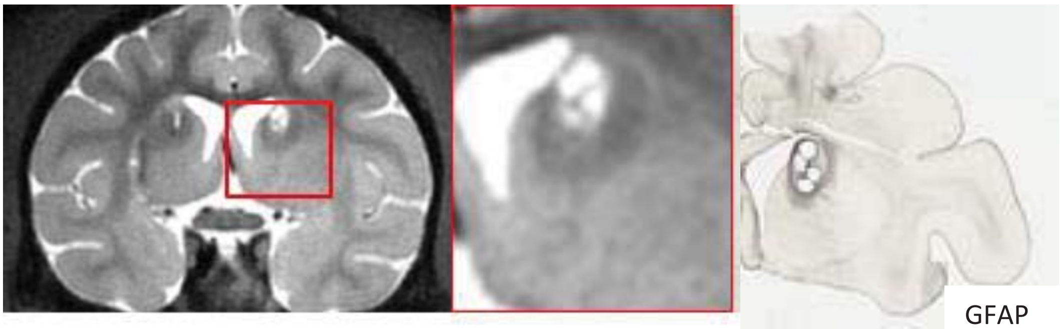

GFAP

These results triggered the present study where we centered the testing paradigm on the autologous grafts (the best possible immunological scenario) as the gold standard, while knowing that mismatched allografts are the worst possible scenario (i.e. no survival in the long term in the brain in the absence of peripheral immunosuppression). This is also the reason why we terminated all mismatched grafted animals at 3 months post-grafting, to prevent the complete loss of the graft that would have precluded the analysis of any immune process possibly underway.

Our data suggests that the "background" inflammation caused by the lesion is not the (only) cause of rejection because autologous grafts are not immunogenic at 3 or 6 months post-grafting while mismatched grafts are heavily attacked in the present study and fully rejected (n=6) in our previous, unpublished study).

In this light, the most important question was to analyze the outcome of matched grafts and compare it to the autologous gold standard. Therefore, we chose to refrain from terminating any

matched animals at an early timepoint and decided to keep them alive to understand the contribution of HLA-matching to long-term survival (6 months being the timepoint at which all our mismatched grafts are rejected). In our view, our design allows us to compare the immunogenicity and survival of matched cells vs autologous cells at a timepoint that is clinically relevant and that cannot be reached when using mismatched cells.

5. The authors should present data on whether the iPSCs are integration free (no random integration of reprogramming vector into the genome). The abnormal expression of reprogramming factors is known to induce immunogenicity of iPSC-derived cells.

We fully agree with the reviewer that it is necessary to exclude an experimental artifact due to the manipulation of the iPSCs to correctly interpret the immune reaction observed in our study.
Sendai virus genome and transgene can sometime remain detectable in freshly reprogrammed iPSCs after limited numbers of passages (e.g<10). In order to exclude this possibility, we performed RT-PCR analysis on Mac1,2,3_iPSC lines to detect the SeV genome and transgenes in cells reprogrammed using the CytoTune™ 2.0 Sendai reprogramming vectors according to the Invitrogen's user guide. We performed end-point RT-PCR with four pairs of primers either targeting all three sendai viruses used during reprogramming (SeV: target amplicon = 181 bp) or targeting each vector individually (KOS: target amplicon = 528 bp specific for CytoTune™ 2.0 KOS, c-Myc: target amplicon of 532 bp specific for CytoTune™ 2.0 hc-Myc, and Klf4: target amplicon = 410 bp specific for CytoTune™ 2.0 hKlf4 vectors). We used human fibroblast cells, 7 days post exposure to those three sendai-viruses used as positive control. No specific amplification at the expected size (Supplementary Fig. 2b) was detected in any of these analyses for Mac1, Mac2 and Mac3_iPSC cells indicating that abnormal expression of reprogramming factors did not induce the immunogenicity of iPSC-derived cells.

We included these results in the supplementary information and in the methods section.

6. Based on previous findings that endothelial cells derived from mouse iPSCs can be immune tolerated by syngeneic recipients due to overexpression of immune suppressive cytokine IL-10, the authors should analyze the expression of immune suppressive cytokines in neural cells derived from iPSCs.

We thank the reviewer for pointing out this line of work. P.E de Almeida and coauthors recently described (de Almeida et al. Nat Comm 2014 (de Almeida, Meyer et al. 2014) the immune response elicited by autologous-iPSC derived endothelial cells in mouse. The authors showed that these cells exhibit long-term survival in vivo and prompt a tolerogenic immune response characterized by elevated IL-10 expression. We investigated whether the induction of the two immuno-suppressive cytokines IL-10 and TGFb1 described in this paper accompanied differentiation of Mac_iPSC into striatal cells (Cell therapy product grafted in Macaques) and could thus contribute to immunological self-tolerance observed in AU context. We compared the expression levels of IL10, TGFb1, DLX2 (a striatal marker) and NANOG (an iPSC marker) by quantitative RT-PCR in iPSC cells (Mac_iPS) and iPSC-derived striatal cells (Mac_CTP). While Almeida et al described an approximately ~50-fold increase of IL10 and ~8 fold-increase during differentiation of iPSC into endothelial cells, we observed no significant increase of IL10 and TGFb1 expression during iPSC striatal differentiation. This suggests that IL10 or TGFb1 mediated immunomodulation by grafted cells is unlikely to occur in our experimental setting in NHP.

We edited the introduction (see below comment #7) to mention this publication and would be willing to include these QRT-PCR results in the supplementary information if you think this is pertinent.

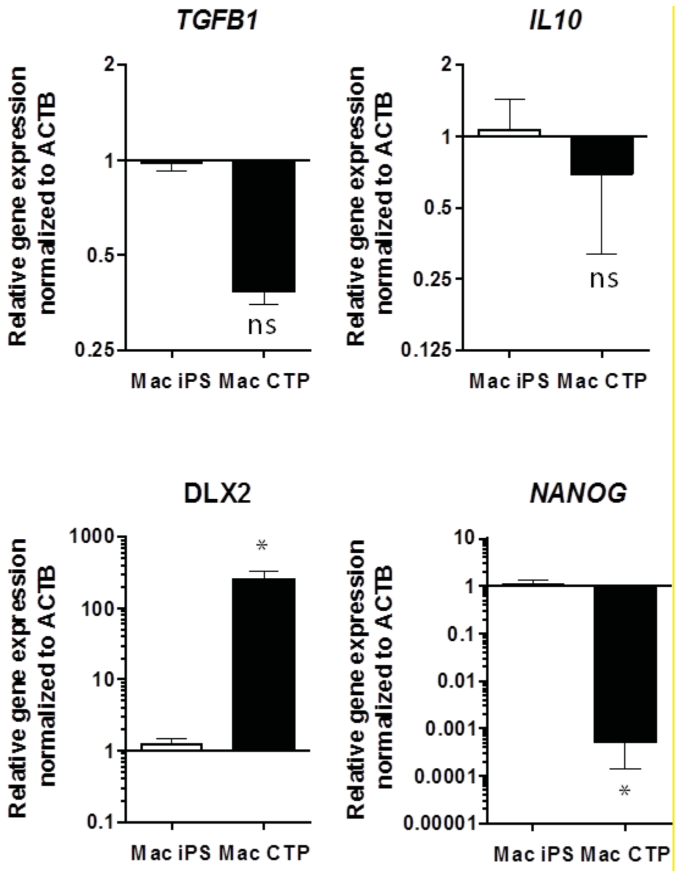

*Figure: QRT-PCR analyses of the expression levels of IL10, TGFb1, DLX2 in iPSC cells (Mac_iPS) and iPSC-derived striatal cells (Mac_CTP) (t-test n=3 lines per group; *p<0.05)*

7. In the introduction, some information on previous studies of the immunogenicity of iPSC-derived cells is incorrect. For example, the humanized mouse studies have shown that certain human iPSC derived cells are robustly immune rejected.

We totally agreed with the point raised by the reviewer. At this stage it appears that such a paragraph was not clear enough and we have rephrased it to clearly share these preexisting observations with the readership. In this light, we have now modified the introduction as follows:

"Some pre-clinical studies using autologous or syngeneic iPSC-derived grafts, the ideal immunological combinations, showed that such grafts can be well tolerated even in non-immune privileged sites in humanized mice and in the non-lesioned brain of non-human primates (NHPs) (Araki, Uda et al. 2013; Morizane, Doi et al. 2013). In contrast, others have reported that mouse and human iPSC derivatives can be immunogenic in syngeneic or autologous recipients and in an autologous humanized mouse model respectively (Zhao, Zhang et al. 2011; de Almeida, Meyer et al. 2014; Zhao, Zhang et al. 2015). Interestingly, it has also been shown that a host immune response (T-cell infiltration) associated with

necrosis following transplantation of syngeneic iPSC, appears to be dependent on the antigenic profile of the transplant (Zhao, Zhang et al. 2011; Kim, Manzar et al. 2017).”

Reviewer #2 (Remarks to the Author):

The study by Badin et al addresses a issue of great current interest in the cell therapy field. Understanding of how host immune responses to iPSC-derived cell products, transplanted to the brain, can be controlled by immune matching will be of great value in the planning of future clinical trials. From that point of view this paper is a valuable and welcome contribution. However, the study has several shortcomings that need to be dealt with before it can be considered for publication.

1. The presentation of the histological data from the transplanted animals needs to be complemented with high power pictures showing the cellular composition of the transplants. From the low magnification pictures shown in Fig 1C it seems that the grafts are quite heterogeneous in composition with large numbers of immature-looking cells, suggesting that they have expanded considerably in size over the 6 months period. For this reason it would be essential to include an analysis of the extent of cellular proliferation at the three time points, 1, 3 and 6 months using eg PCNA staining. This is important since continuously expanding grafts are likely to contain areas where the BBB remains immature and leaky. Similarly, the MR images in Fig 1B are too small to allow the reader to evaluate the images. The relevant hyper-intense areas should be indicated.

It is notable that grafts are placed in both caudate and putamen (as mentioned in Methods), but this is not properly commented on in the results section. They should be independently labeled in the figures.

We thank the reviewer for raising this very important point that has taken us to perform new post-mortem analyses to understand the potential contribution of cell proliferation to the immunogenicity of the grafts.
Figure 1c has now been modified to clearly show high magnification pictures of stainings with different markers in grafts positioned both in the caudate and the putamen.
As you can see from these pictures, FOXG1 and Calretinin staining account for most of the cells in the graft showing their telencephalic nature.
The staining and quantification of a proliferation marker, PHH3 (Phospho-histone H3), has now been performed and shows that less than 1/10.00 cell (<0.1%) in the graft are actively dividing at 3 months post-grafting and this ratio is even significantly lower (<0.02%) at 6 months post-grafting. This quantification is reported below (AU_NHP: white circle and square, MI_NHP:black circle, MA_NHP: grey square) and we would be happy to include it as supplementary data if you think it will improve the interpretation of our results and the impact of our findings.

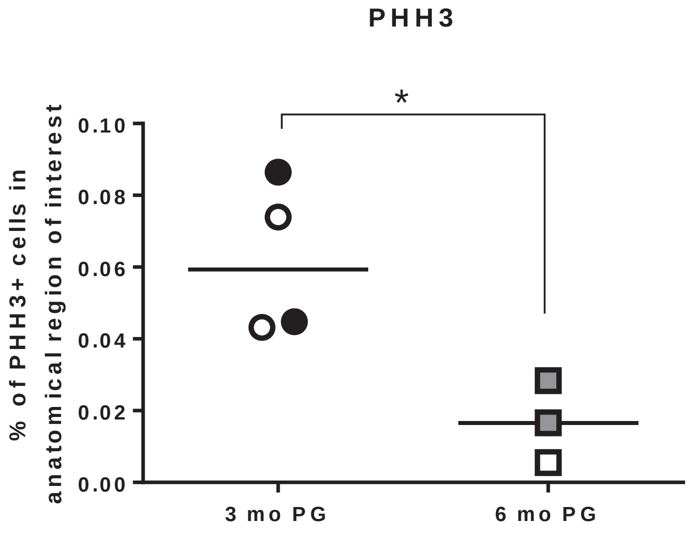

Moreover, we have performed double staining of PHH3 and different antibodies to exclude that these small fractions of proliferating cells were immune cells. Results were negative for GFAP, CD45, CD68, HLA-DR, CD4 and CD8.

Figure 1b has now been modified to include red arrows indicating the white, hyperintense areas associated with CSF infiltration of vacuolated grafts.

2. It is important that the procedure used to generate the MHC matched cells is described in detail. I am not an immunologist, but I find it unsatisfactory that the exact description of which MHC genes/Mafa haplotypes have been selected for matching is lacking, and whether the resultant cell line was homozygous for these genes.

In response to this comment we now provide a more detailed description of MaFaLA haplotypes of donor and recipient NHP used in the study. Briefly, the MHC genotype was determined by genotyping 17 microsatellites scattered across the MHC region as previously described in Blancher et al 2012 (Blancher, Aarnink et al. 2012).

Figure 1 has been modified to more accurately describe the precise haplotype of each donor and recipient NHP that were coded according to published data on the seven mauritian cynomolgus MHC ancestral haplotypes. In parallel, the supplementary table 1 and the text were corrected to reflect thees changes in nomenclature and provide additional and clearer information to the reader. Furthermore, we have improved the methods section and added a new Supplementary Figure 1_R1 to show the exact size measured for each of the 17 microsatellites genotyped in all donor and recipient NHPs of the study.

The correspondence between the Mafa haplotype nomenclature used in the Morizane paper and ours is available in Wiseman et al 2013 (Wiseman, Karl et al. 2013) and has now been included in the reference list.

3. The discussion fails to deal with several issues of importance for the interpretation of the reported (negative) results. In particular, the differences between the extent of MHC matching in this study

and the one used in the recent Morizane et al paper need to be discussed in detail. Were the same Mafa genes targeted for matching in both studies, and to what extent did the Mafa haplotypes match the ones expressed by the monkeys used in the transplantation experiment in this study? (In the Morizane study the recipient monkeys had at least one of the alleles identical to the MHC matched transplants). To make the discussion really interesting, the authors should include a discussion of the shortcoming(s) of the degree of MHC matching used in this study, and how it could be improved in order to obtain graft cells that are sufficiently well matched to evade the immune response. A detailed comparison with the procedure and matching used in the Japanese study may shed light on this interesting question.

We thank the reviewer for raising this very important point. We have looked in detail at the matching presented in the Morizane et al. paper (supplementary table 1) and even if the techniques used in both studies differ, a table of correspondence has been published, and we found that our results are globally similar to theirs. The same Mafa genes were indeed targeted in both studies: class IA, IB and Class II DR, DQ, DP. The supplementary data added in response to question 2 now illustrates that in our samples we have obtained 100% matching of in all MA recipients as the donor is homozygous and its haplotype is shared with the recipient. This was also the case for matched NHP recipients in the Morizane study. Moreover, it is important to note that in the particular case of Mauritian cynomolgus macaques, the microsatellite typing panel that spans the entire 5Mb MHC region (class I and II) allows to infer the complement of MHC alleles expressed by the subject as accurately as the deep sequencing technique (Wiseman, Karl et al. 2013). .

4. Minor comment: In the discussion of the reasons for the different results obtained in this and the Morizane et al study I suggest that the authors should add one more factor that may be of particular importance, namely that grafts that expand in size after transplantation, due to continued cell division of more immature cells, may retain areas with immature or leaky BBB and thus be more prone to trigger the host's immune response.

We thank the reviewer for this additional consideration that we have added to widen the discussion.

Reviewer #3 (Remarks to the Author):

This is a difficult paper to evaluate. Firstly these are difficult to perform experiments that often will never be repeated and thus the data provide value to the scientific community. However the study is difficult to interpret within the context of grafting of striatal IPSC's for Huntington's disease. Prior studies, as acknowledged by the authors demonstrate successful grafting with unlesioned animals. The present study performed similar grafting in quinolinic acid lesioned monkeys. The question is, how representative is that in terms of the necrotic environment the QA lesions causes to that of HD victims. The answer is the QA lesion provides a far more violent environment than what is seen in HD with liquifactive necrosis, holes in the tissue and robust inflammation. Therefore is this model a fair test for the question under study. I wonder if the same result would be found with a more gentle lesion such as the other lesioning methods this outstanding group has published in the past.

We warmly thank the reviewer for recognizing the value of the data generated and appreciating the difficulty in performing NHP studies.

We fully agree with the reviewer that exitotoxic lesion models can be extremely severe. However, for the purpose of grafting studies, we have changed the concentration of QA usually used and published by our group (180mM). In fact, pilot studies were performed to determine the concentration of QA that produced a glial scar but no vacuolation/liquifactive necrosis. We also studied the window to graft cells after lesioning in order to promote implantation. The quantifications of additional inflammatory markers now added in the revised Fig.1_R1 and in a new supplementary Fig. 6 also show the extent of macrophages and T lymphocytes found in QA-lesioned controls. The precise characterisation of this 80mM QA lesion model in terms of NeuN loss in the striation and input and output structures as well as behavioural and PET (FDG, Fallypride) deficits is the object of a separate scientific paper that is currently under revision for publication in Neurobiology of Disease Journal.

Overall the data suggest that the model reproduces a late stage HD, presenting MSN loss and a significant astrocytic inflammation that can be pertinent to the clinical context. Our laboratory has also been developing a primate model of HD by overexpressing mutant Huntingtin via viral vectors but no neuronal dysfunction or loss has been observed at 18 months post-injection (unpublished data). In the absence of phenotypic and pathological deficits we consider the model to prodromal to use it to validate the potential contribution of cell replacement therapies at this stage.

Refrences

Araki, R., M. Uda, et al. (2013). "Negligible immunogenicity of terminally differentiated cells derived from induced pluripotent or embryonic stem cells." Nature **494**(7435): 100-104.

Blancher, A., A. Aarnink, et al. (2012). "Use of Cumulative Poisson Probability Distribution as an Estimator of the Recombination Rate in an Expanding Population: Example of the Macaca fascicularis Major Histocompatibility Complex." G3 (Bethesda) **2**(1): 123-130.

de Almeida, P. E., E. H. Meyer, et al. (2014). "Transplanted terminally differentiated induced pluripotent stem cells are accepted by immune mechanisms similar to self-tolerance." Nat Commun **5**: 3903.

Emborg, M. E., Y. Liu, et al. (2013). "Induced pluripotent stem cell-derived neural cells survive and mature in the nonhuman primate brain." Cell Rep **3**(3): 646-650.

Emborg, M. E., Z. Zhang, et al. (2013). "Intracerebral transplantation of differentiated human embryonic stem cells to hemiparkinsonian monkeys." Cell Transplant **22**(5): 831-838.

Kikuchi, T., A. Morizane, et al. (2017). "Human iPS cell-derived dopaminergic neurons function in a primate Parkinson's disease model." Nature **548**(7669): 592-596.

Kim, E. M., G. Manzar, et al. (2017). "Induced pluripotent stem cell-derived gamete-associated proteins incite rejection of induced pluripotent stem cells in syngeneic mice." Immunology **151**(2): 191-197.

Kriks, S., J. W. Shim, et al. (2011). "Dopamine neurons derived from human ES cells efficiently engraft in animal models of Parkinson's disease." Nature **480**(7378): 547-551.

Morizane, A., D. Doi, et al. (2013). "Direct comparison of autologous and allogeneic transplantation of iPSC-derived neural cells in the brain of a non-human primate." Stem Cell Reports **1**(4): 283-292.

Morizane, A., T. Kikuchi, et al. (2017). "MHC matching improves engraftment of iPSC-derived neurons in non-human primates." Nat Commun **8**(1): 385.

Sugita, S., K. Makabe, et al. (2017). "Detection of Retinal Pigment Epithelium-Specific Antibody in iPSC-Derived Retinal Pigment Epithelium Transplantation Models." Stem Cell Reports **9**(5): 1501-1515.

Wiseman, R. W., J. A. Karl, et al. (2013). "Haplessly hoping: macaque major histocompatibility complex made easy." ILAR J **54**(2): 196-210.

Zhao, T., Z. N. Zhang, et al. (2011). "Immunogenicity of induced pluripotent stem cells." Nature **474**(7350): 212-215.

Zhao, T., Z. N. Zhang, et al. (2015). "Humanized Mice Reveal Differential Immunogenicity of Cells Derived from Autologous Induced Pluripotent Stem Cells." Cell Stem Cell **17**(3): 353-359.

Reviewers' comments:

Reviewer #1 (Remarks to the Author):

The authors have addressed the concerns of this reviewer. Please include the data on the expression of IL10 and TGFb of differentiated cells in the supplementary figure.

Reviewer #2 (Remarks to the Author):

I think this revised version is improved in several respects. The background literature as well as the the discussion of the results are now dealt with in a good way. However, the reporting and documentation of the cellular composition of the grafts are still poor and unsatisfactory. The two cellular markers reported in Figure 1 b and c are rather uninformative and the pictures are displayed in a miniature format that makes it difficult to evaluate them. These pictures suggest that these grafts remain immature and are largely composed of densely packed immature progenitors. This is an important point and could be one factor that explains the activation of host´s immune response. To clarify this point the authors need to perform and report staining with antibodies that recognise more mature, postmitotic neurons, such as NeuN, Map2 and DARPP32, and also include additional markers of proliferative forebrain progenitors, such as nestin. The authors should be asked to prepare a separate figure where these pictures, together with the ones now shown in Figure 1a and b, are displayed in a larger format, and they should also expand the description of cell composition in the main text.
The authors have chosen to use PHH3 as proliferative marker. This is a relatively new marker and this reviewer has so far not seen it applied to proliferative neural transplants (in contrast to the more commonly used markers PCNA and Ki67). For this reason I think it would be valuable to include a picture documenting the staining quality and the phenotypic identity of the cells stained with the PHH3 marker.

Reviewer #3 (Remarks to the Author):

First I apologize to the authors for my tardy review. I remain skeptical as to the utility of this model to address the question posed but the authors response is adequate although I do not necessarily agree that the level of QA toxicity in the model is simnilar ot ewnd-stage HD. I stand by my original comment that the model is overly severe to model what is going on in HD, but I think the value of the data is significant and these experiments are likely not to be repeated. However, the authors should comment on this aspect of the model in the discussion. The reader will be able to interpret the data as they wish. Furthermore I believe they have responded well to the other reviewers comments, adding valuable data.

POINT-BY-POINT RESPONSE

Reviewer #1 (Remarks to the Author):

The authors have addressed the concerns of this reviewer. Please include the data on the expression of IL10 and TGFb of differentiated cells in the supplementary figure.

We thank the reviewer for considering our work suitable for publication. As requested, we have now included in Supplementary figure 4c the data on IL10 and TGFb expression in differentiated cells.

Reviewer #2 (Remarks to the Author):

I think this revised version is improved in several respects. The background literature as well as the the discussion of the results are now dealt with in a good way. However, the reporting and documentation of the cellular composition of the grafts are still poor and unsatisfactory. The two cellular markers reported in Figure 1 b and c are rather uninformative and the pictures are displayed in a miniature format that makes it difficult to evaluate them. These pictures suggest that these grafts remain immature and are largely composed of densely packed immature progenitors. This is an important point and could be one factor that explains the activation of host´s immune response. To clarify this point the authors need to perform and report staining with antibodies that recognise more mature, postmitotic neurons, such as NeuN, Map2 and DARPP32, and also include additional markers of proliferative forebrain progenitors, such as nestin. The authors should be asked to prepare a separate figure where these pictures, together with the ones now shown in Figure 1a and b, are displayed in a larger format, and they should also expand the description of cell composition in the main text.

We agree with reviewer #2 that the cellular identity, maturity and proliferative status of our grafts in each type of NHP recipients are important to explain the activation of the host's immune response. In response to reviewer #2's comments, we have modified Fig1 b&c and created 4 new supplementary figures to expand the description of the cell composition of the graft.
In order to address the first point raised regarding the size of the images, we have changed Figures 1 b&c to show larger and higher magnification images of the graft in MI, AU (now showing Mac2) and MA recipients.
To address the second issue raised regarding the maturity of the grafted cells in relation to the host's immune response, we have created four new supplementary figures to illustrate the neuronal identity and the degree of maturity of the different grafts at low (Supplementary Figures 6 and 8) and high magnification (Supplementary Figures 7 and 9), respectively. As suggested by the reviewer, we have used three classical neuronal markers of post-mitotic neurons that stain the nuclei (NeuN), peri-nuclear soma (HuC/D) and soma, and neuritic extensions (MAP2) to demonstrate that the vast majority of graft-derived cells are post-mitotic neurons. The high magnification pictures included in Supplementary Figure 7 better document the cellular morphology of graft-derived cells.
We used SOX1 to stain immature neural cells as it has been reported that nestin can be expressed by host glial cells in a neuro-inflammatory context as it is the case here due to the QA lesion (Choi, Riew et al. 2017). Indeed, in their study Choi et al could demonstrate that desmin, GFAP, and nestin have characteristic time-dependent expression patterns in reactive astrocytes in 3NP striatal lesioned rats. Similar observations were made by Krishnasamy and colleagues in activated microglia (Krishnasamy, Weng et al. 2017).

We used PHH3 to unequivocally stain proliferative cells in the core of the graft. Both SOX1 and PHH3 staining show that immature cells are in fact rarely detected in recipients at 6 months post-grafting in AU or MA recipients. Not surprisingly, and as mentioned in the previous point-by-point response and in the R1_manuscript, the number of proliferative/immature cells in NHP recipients is significantly higher at 3 months post-grafting than at 6 months (although overall at less than 0.02% of all graft-derived cells). This quantitative result was confirmed comparing SOX1 staining of AU recipients at 3 and 6 months post-transplantation (see figure 1 below that can be included as a supplementary figure at the reviewer's discretion). As expected, the vast majority of graft-derived cells are initially striatal neuronal precursors that mature into post-mitotic neurons within 3 months. A very small (<0.02%) proportion likely remains immature up to, and possibly beyond, 6 months post-grafting.
We agree with Reviewer #2 that, even if transient, immaturity of graft-derived cells is one of the factors that may contribute to trigger the host immune response. However, our results suggest that this effect is time-dependent and not treatment group-dependent. Therefore, cell immaturity does not by itself explain the difference in immune response triggered by AU and MA grafts at the same timepoints.
We have now added to the text a new paragraph discussing this issue specifically and suggesting the proliferative status of graft-derived cells as one of the hypothetical variables that may explain the discrepancies between our findings and those previously published (page 7, line 18).

"At least four hypotheses may explain the discrepancies between our findings and those previously published. […]. Thirdly, the antigenic profile, closely related to the maturation time indispensable to generate fully differentiated striatal iPSC-derived neurons, may substantially differ from that of dopaminergic neurons. This may ultimately result in a different, possibly more aggressive, anti-graft immune response. In all cases, our post-mortem data suggest that grafts are largely composed of post-mitotic striatal neurons and that maturation is time-dependent rather than dependent on the recipient-donor MHC matching combination (AU, MA, MI)."

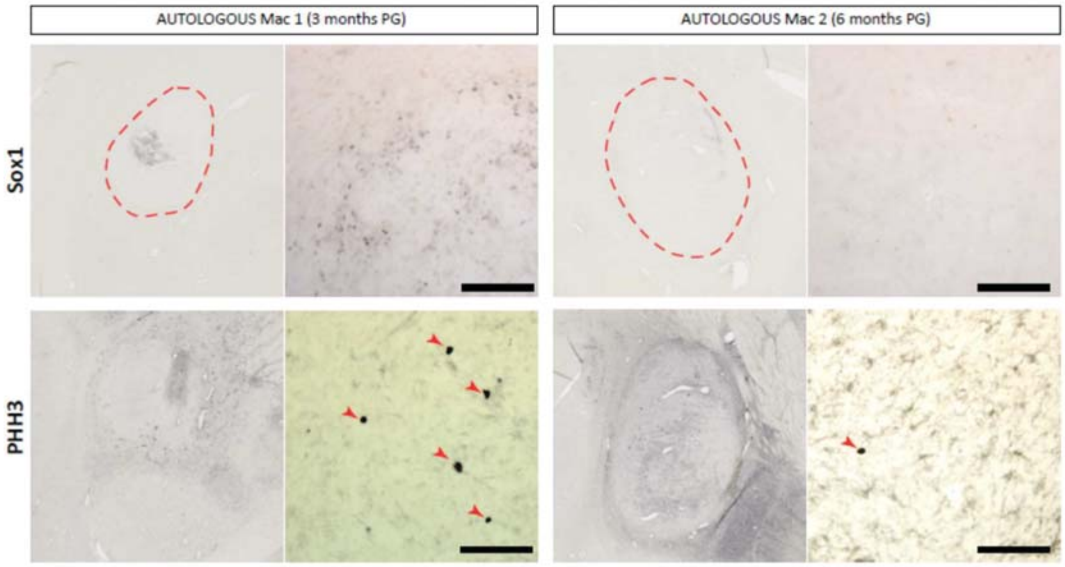

Figure 1: Representative immunostainings showing SOX1 (neural cells) and PHH3 (mitotic cells) of autologous grafts at 3 months PG and 6 months PG). Scale bar: 100 µm.

Finally, we have added supplementary data on the characterization of the striatal identity of grafted cells in relation to the presence of immune cells at low and high magnification (Supplementary Figures 8 and 9, respectively).
We used FOXG1 as a telencephalic marker, Calretinin as a marker of interneurons, and Calbindin as a marker of projection neurons. In parallel we showed that Mafa-DR (using HLA-DR antibodies) was upregulated in the core of the graft, most likely due to immune infiltration although activated glial cells can also display a positive HLA-DR signal. The vast majority of graft-derived cells appear to be CalRet+ striatal interneurons. We detected limited amount of CalB+ striatal neurons and no DARPP32+ neurons. We are currently working on our differentiation protocol for Mac_iPS to generate cell therapy products that would yield preferentially CalB+ or DARPP32+ striatal neurons. This would likely be necessary for optimal striatal repair after a QA-lesion but we believe that our current protocol is still relevant to study the immunogenicity of allogenic striatal grafts in NHP.

The authors have chosen to use PHH3 as proliferative marker. This is a relatively new marker and this reviewer has so far not seen it applied to proliferative neural transplants (in contrast to the more commonly used markers PCNA and Ki67). For this reason I think it would be valuable to include a picture documenting the staining quality and the phenotypic identity of the cells stained with the PHH3 marker.

We agree with the reviewer that, to date, this antibody has not been applied to the neuroscience field but only to the oncology field. As recently discussed by Elmaci et al., Histone H3 phosphorylation on serine-10 is specific to mitosis and phosphorylated histone H3 (PHH3) proliferation markers (as counts per cell numbers) are increasingly being used to evaluate proliferation in various tumors (Elmaci, Altinoz et al. 2018). When comparing PHH3 and Ki67 mitotic counts of neuroendocrine tumors, Voss et al (Voss, Riley et al. 2015) found that mitotic counts by PHH3 and Ki67 significantly correlated and that inter-pathologist correlation was best for PHH3 counts. Likewise, Gerring's et al. head-to-head comparison of Ki67 and phosphohistone H3 showed that phosphohistone H3 outperforms Ki67 (Gerring, Pearson et al. 2015). Like many authors in the cancer field, we found that PHH3-staining highlights mitotic cells showing a virtually ON/OFF signal which makes the quantification of mitotically active areas easier and more unequivocal compared to continuous, and thus more ambiguous, Ki67 or PCNA staining.

We believe that the use of this antibody is appropriate in the neuroscience field and, as requested by the reviewer, we have included pictures documenting the PHH3 staining quality at low and high magnification in Supplementary Figures 6 & 7.

Reviewer #3 (Remarks to the Author):

First I apologize to the authors for my tardy review. I remain skeptical as to the utility of this model to address the question posed but the authors response is adequate although I do not necessarily agree that the level of QA toxicity in the model is similar to end-stage HD. I stand by my original comment that the model is overly severe to model what is going on in HD, but I think the value of

the data is significant and these experiments are likely not to be repeated. However, the authors should comment on this aspect of the model in the discussion. The reader will be able to interpret the data as they wish. Furthermore I believe they have responded well to the other reviewers comments, adding valuable data.

As discussed in our previous point-by-point reply, we fully agree with reviewer #3 that exitotoxic lesion models can be severe. However, for the purpose of grafting studies, we have lowered the concentration of QA used and performed pilot studies to demonstrate that our lowered concentration of QA produce a glial scar but no vacuolation/liquifactive necrosis. Figure 2 and Supplementary figure 10 show the extent of macrophage and T lymphocyte infiltration found in QA-lesioned controls. The precise characterization of this 80mM QA lesion model in terms of NeuN loss in the striatum and input and output structures, as well as behavioral and PET (FDG, Fallypride) deficits is the object of a separate scientific paper that has just been accepted for publication in Neurobiology of Disease Journal(Lavisse, Williams et al. 2019). We can only agree with the reviewer that all models of human disease are limited and partial. Overall, our data shows that the model reproduces some of the important features of HD, such as MSN loss and a significant astrocytic inflammation that, in the absence of better large animal models, appear pertinent to test therapeutic strategies in the preclinical context. In addition, in order to investigate the role of QA inflammation, we included a group of non-transplanted NHP exclusively exposed to QA treatment.

As requested by the reviewer, we have added a sentence in the discussion to acknowledge the limitations of this animal model and allow the reader to interpret the data accordingly (page 7, line 13).

"Indeed, even though acute excitotoxic lesions may bear only limited resemblance to the chronic and progressive inflammation observed in HD patients, transplanting into a lesioned brain may be more pertinent as it more closely mimics the inflammatory milieu associated with neurodegenerative disorders in man."

References

Choi, J. H., T. R. Riew, et al. (2017). "Desmin expression profile in reactive astrocytes in the 3-nitropropionic acid-lesioned striatum of rat: Characterization and comparison with glial fibrillary acidic protein and nestin." Acta Histochem **119**(8): 795-803.

Elmaci, I., M. A. Altinoz, et al. (2018). "Phosphorylated Histone H3 (PHH3) as a Novel Cell Proliferation Marker and Prognosticator for Meningeal Tumors: A Short Review." Appl Immunohistochem Mol Morphol **26**(9): 627-631.

Gerring, Z., J. F. Pearson, et al. (2015). "Phosphohistone H3 outperforms Ki67 as a marker of outcome for breast cancer patients." Histopathology **67**(4): 538-547.

Krishnasamy, S., Y. C. Weng, et al. (2017). "Molecular imaging of nestin in neuroinflammatory conditions reveals marked signal induction in activated microglia." J Neuroinflammation **14**(1): 45.

Lavisse, S., S. Williams, et al. (2019). "Longitudinal characterization of cognitive and motor deficits in an excitotoxic lesion model of striatal dysfunction in non-human primates." Neurobiol Dis: 104484.

Voss, S. M., M. P. Riley, et al. (2015). "Mitotic count by phosphohistone H3 immunohistochemical staining predicts survival and improves interobserver reproducibility in well-differentiated neuroendocrine tumors of the pancreas." Am J Surg Pathol **39**(1): 13-24.

REVIEWERS' COMMENTS:

Reviewer #2 (Remarks to the Author):

I find that the authors have addressed my concerns in a good way. The documentation of the cellular composition of the grafts is a valuable addition to the paper.
